# Supplementary material for: Modeling short visual events through the BOLD moments video fMRI dataset and metadata
Source: Nat Commun. 2024 Jul 24;15:6241. doi: 10.1038/s41467-024-50310-3 (PMC11269733; doi:10.1038/s41467-024-50310-3)
Supplement: Supplementary file 1 — Supplementary Information [file 41467_2024_50310_MOESM1_ESM.pdf]

Modeling short visual events through the BOLD Moments video fMRI dataset and metadata

Supplementary material to the main manuscript

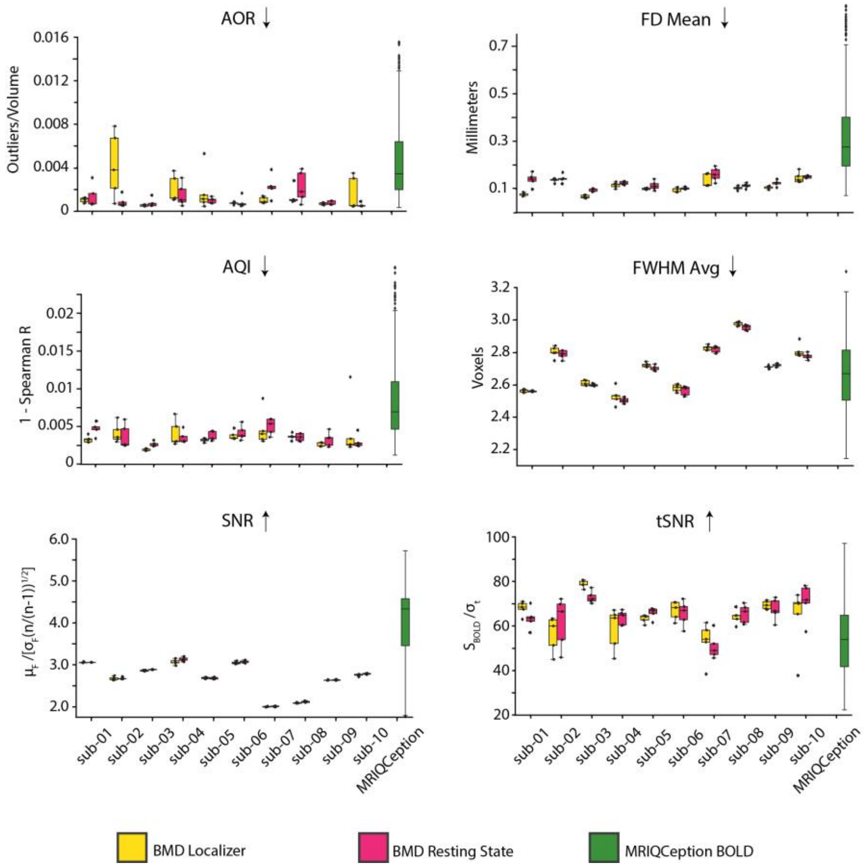

Supplementary Figure 1. **Localizer and resting state functional scan data quality.** Panels compare IQM values for each subject for the localizer and resting state functional runs in the BMD (per subject: localizer, n=5; resting state, n=5) with anonymous BOLD data from MRIQCeption (BOLD, n=624). Source data are provided as a Source Data file. The overlaid points correspond to the IQM value for the BMD subject's individual run. MRIQCeption does not distinguish between different tasks within BOLD scans. The boxplot extends 1.5 times the high and low quartiles, with outliers defined as a scan with a value outside that range and denoted by diamonds. The up or down arrows after the IQM title correspond to whether higher or lower IQM values denote higher data quality. X-axis labels are shared vertically.

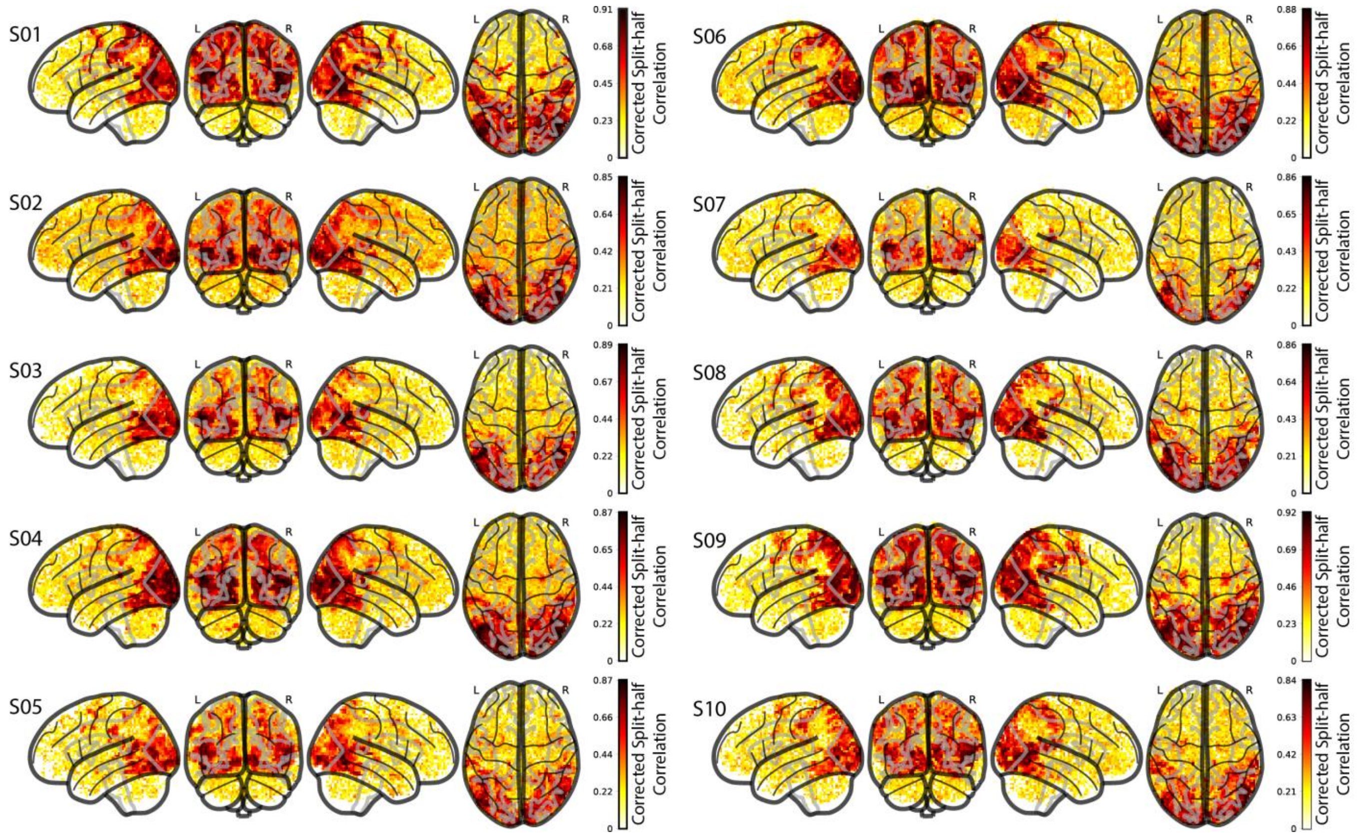

Supplementary Figure 2. **Whole-brain split-half reliability for all subjects.** The glass brains show the split-half reliability (Spearman Brown corrected) at every voxel for each of the ten subjects. A Pearson's R correlation value was obtained by correlating random splits of the 10 repetitions from the 102 testing videos. The Spearman Brown split-half reliability was computed using the Pearson's R (ρ) value from the formula: Spearman Brown = ($2\rho/(1+\rho)$), equation (1).

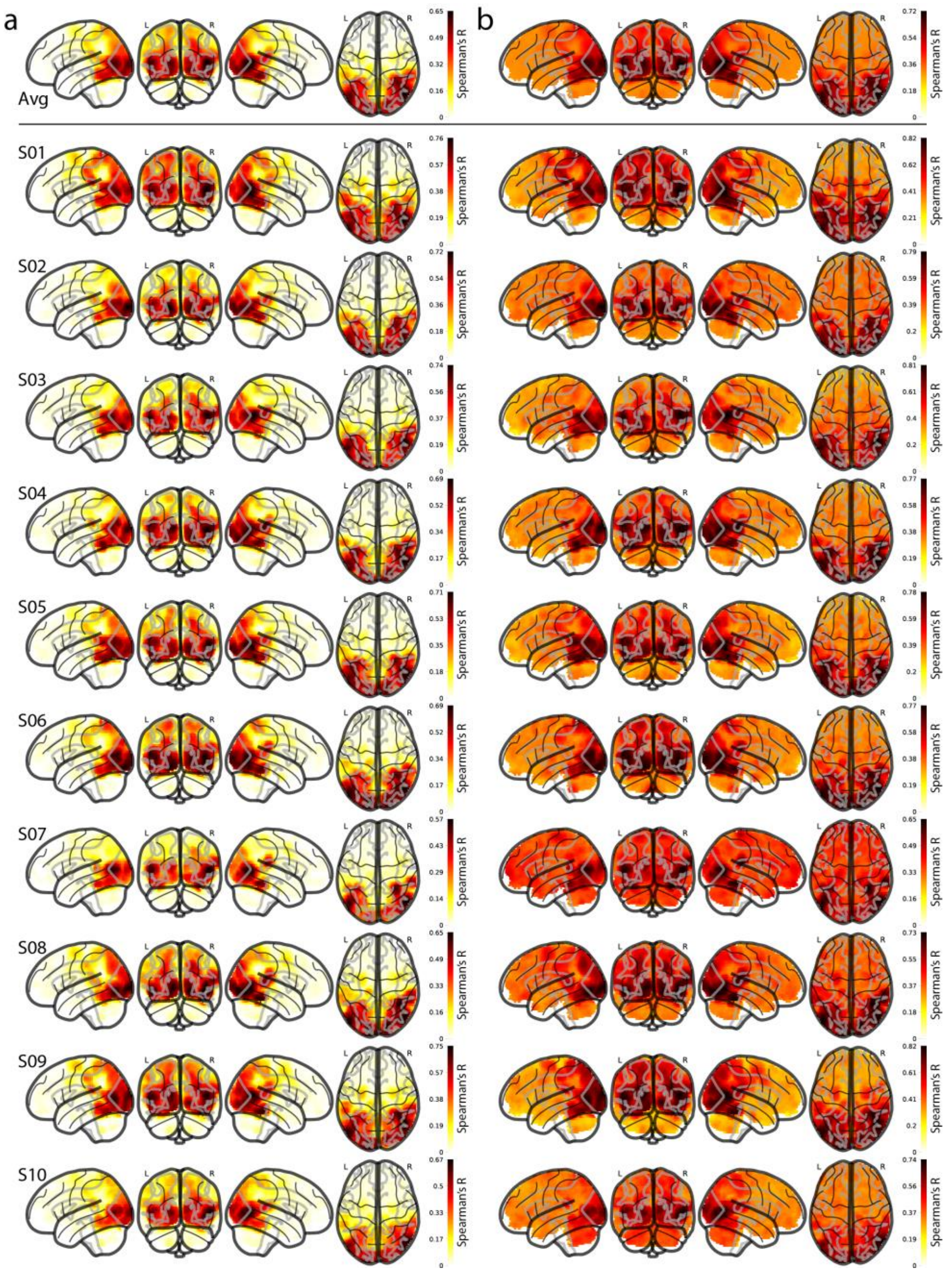

Supplementary Figure 3. **Whole-brain multivariate searchlight-based noise ceilings for all subjects. a** Lower noise ceiling: The lower noise ceiling is estimated using a leave-one-subject out procedure. A subject's RDM at a given voxel *v* is correlated (Spearman's R) with the remaining nine-subject average RDM at that voxel *v*, repeated over all voxels. **b** Upper noise ceiling: The upper noise ceiling is estimated by correlating (Spearman's R) a subject's RDM at a given voxel *v* with the ten-subject group average RDM at that voxel *v*, repeated over all voxels. We show the whole-brain visualization for the upper and lower noise ceilings averaged over all subjects (top row) and each subject individually (bottom). RDMs are computed from the testing set.

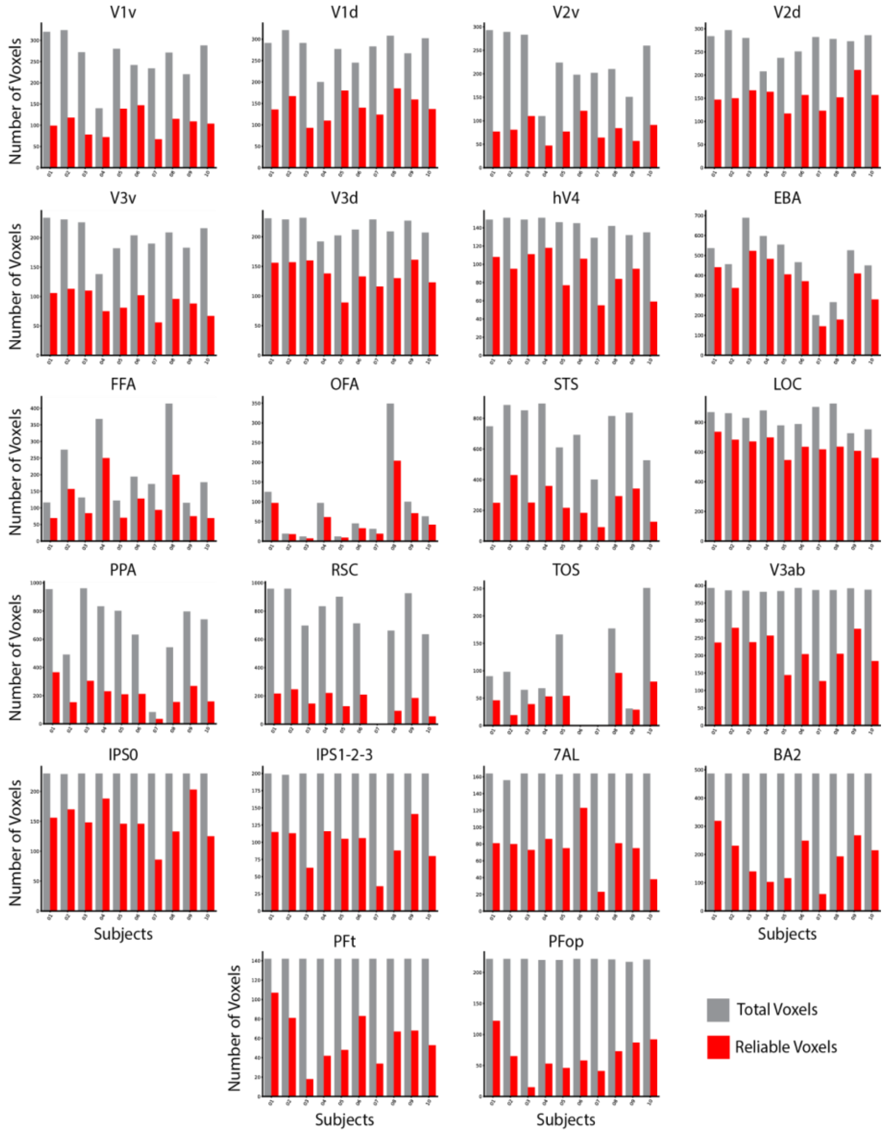

Supplementary Figure 4. **ROI reliability and size for each subject.** The bar plots show the total number of voxels in the ROI mask (gray bar) and the total number of reliable voxels ($p<0.05$, Spearman-Brown) in the ROI mask (red bar) for each subject across the 22 ROIs. Subject 6 did not show any activation from the functional localizer task for ROI transverse occipital sulcus (TOS), and subject 7 did not show any activation from the functional localizer task for ROIs retrosplenial cortex (RSC) and transverse occipital sulcus (TOS). Y-axis and X-axis labels are shared horizontally and vertically, respectively. Source data are provided as a Source Data file.

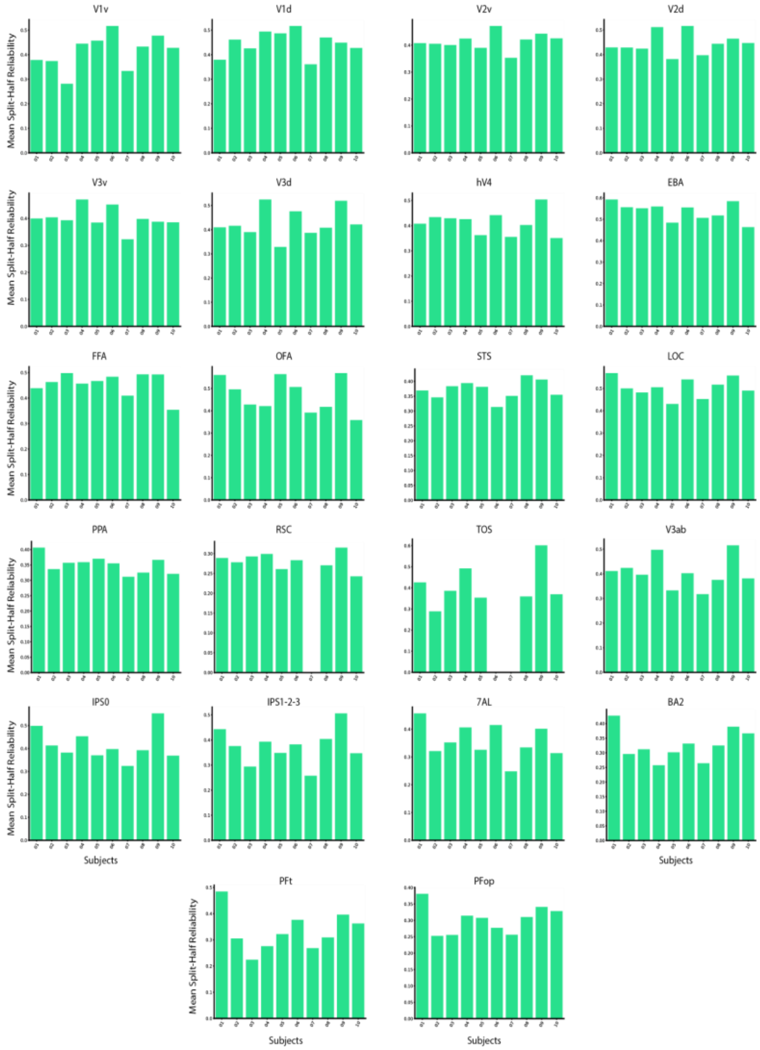

Supplementary Figure 5. **Average reliability in each ROI for each subject.** The bar plots show the average split-half reliability (Spearman Brown corrected split-half correlation) in each ROI of all reliable voxels, separated for each subject. Y-axis and X-axis labels are shared horizontally and vertically, respectively. Source data are provided as a Source Data file.

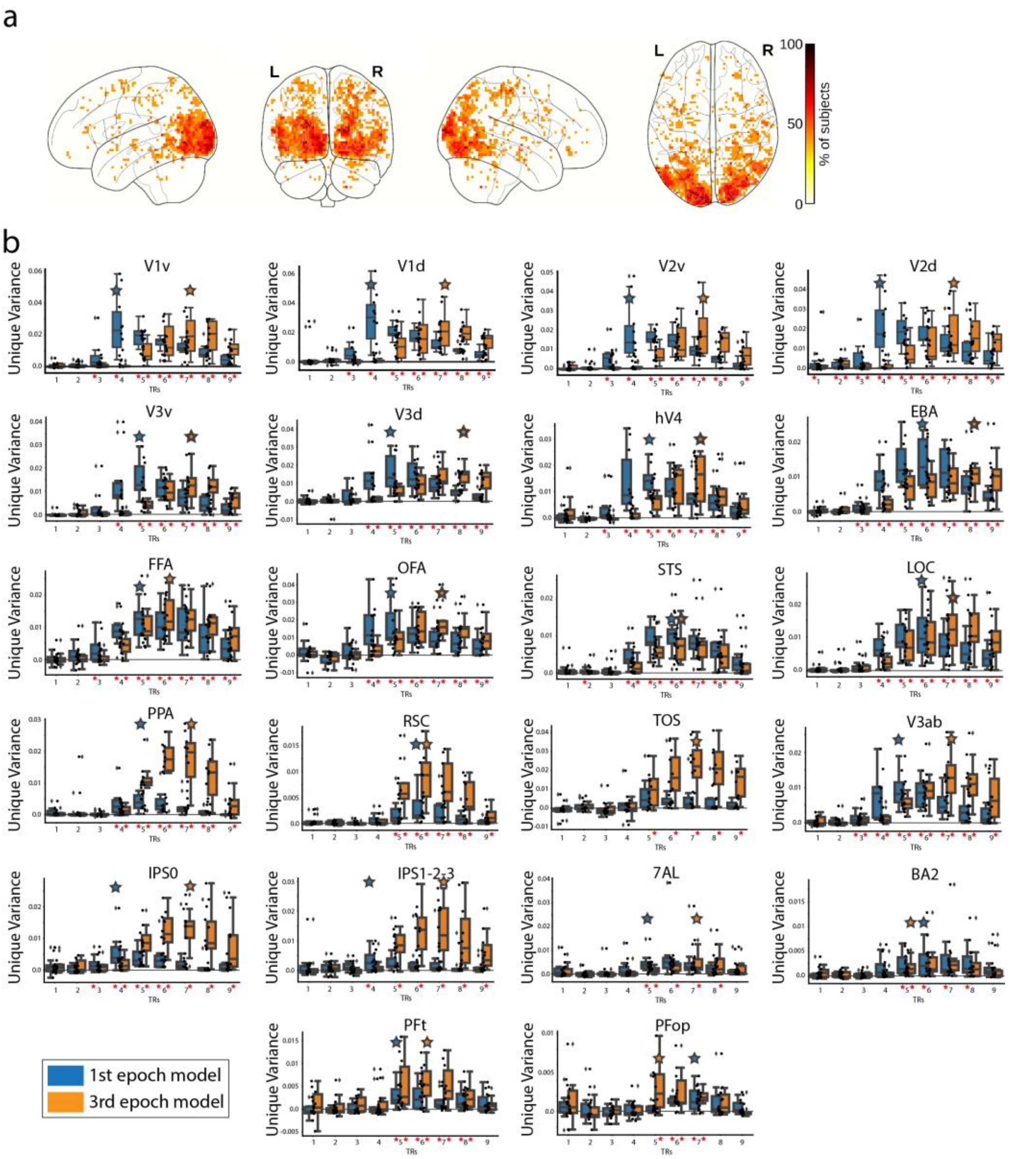

Supplementary Figure 6. **Encoding the temporal dynamics of the BOLD signal. a** Whole-brain analysis: Each voxel shows the percentage of subjects with a TR peak difference of 2 TRs at that specific voxel. Only significant voxels are plotted (p<0.05, binomial test, FDR corrected). The TR shifts are observed predominantly in the visual cortex. **b** ROI analysis: Unique variance explained by the first (0-1s) and third video epoch (2-3s) synthetic fMRI data, at each TR. Red asterisks along the x-axis indicate unique variance scores significantly greater than 0 (p<0.05, one-sample one-side t-test, FDR corrected across 9 TRs x 2 video epochs = 18 comparisons). Large blue/orange stars indicate the TR with the highest subject averaged unique variance for the first/third video epochs, respectively. Source data are provided as a Source Data file. The box plot encompasses the first and third data quartiles and the median (horizontal line). The whiskers extend to the minimum and maximum values within 1.5 times the interquartile range, and values falling outside that range are considered outliers (denoted by a diamond). The overlaid points show the value at each observation (n=10 for all ROIs except transverse occipital sulcus (TOS, n=8) and retrosplenial cortex (RSC, n=9)). Y-axis and X-axis labels are shared horizontally and vertically, respectively.

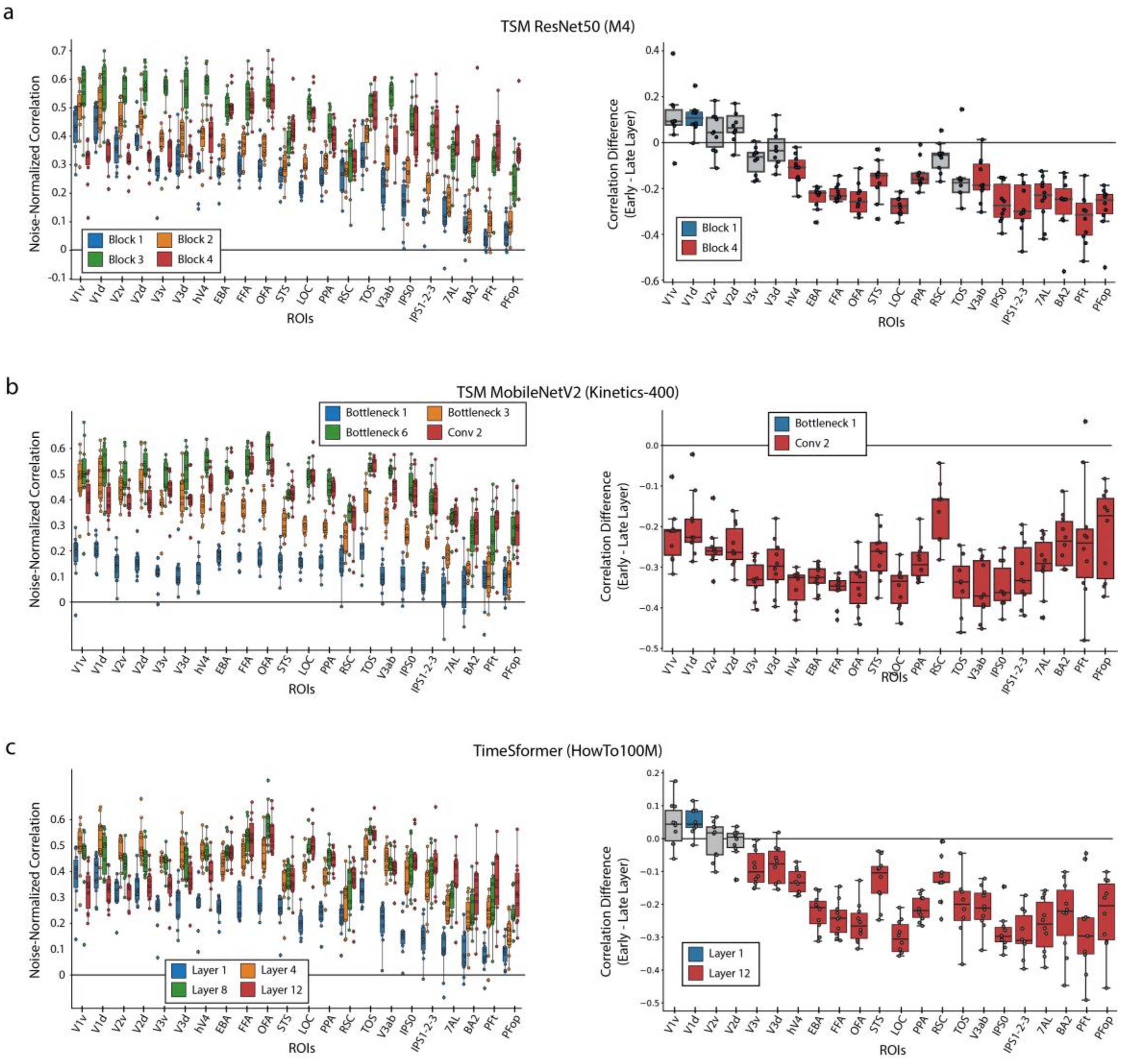

Supplementary Figure 7. **Encoding model performance on BMD**. **a** TSM ResNet50 trained on M4: Features were extracted after blocks 1 (blue), 2 (orange), 3 (green), and 4 (red) in the ResNet 50 architecture. **b** TSM MobileNetV2 trained on Kinetics-400: Features were extracted after the first bottleneck layer (blue), third bottleneck layer (orange), sixth bottleneck layer (green), and last 2D convolutional layer before the average pool (red) in the MobileNetV2 architecture. **c** TimeSformer S+T trained on HowTo100M: Of the twelve model layers, features were extracted after the first (blue), fourth (orange), eighth (green), and twelfth (red) layers. The box plot on the left side in each panel shows the noise-normalized predictivity of four of each architecture's features at each of the 22 ROIs. The features were extracted at early (blue), intermediate (orange and green), and late (red) processing stages in each architecture to capture increasingly high-level degrees of transformations. The box plot on the right side in each panel shows the brain prediction difference between each architecture's most deep and early layers for each subject and ROI. Source data are provided as a Source Data file for all three panels and the left and right graphs. For the box plots on the right, a blue or red colored box plot denotes a significant difference in correlations from 0 ($p<0.05$, two-sided one-sample t-test, Bonferroni corrected for $n=22$ comparisons), and gray denotes no significance. The box plots encompass the first and third data quartiles and the median (horizontal line). The whiskers extend to the minimum and maximum values within 1.5 times the interquartile range, and values falling outside that range are considered outliers (denoted by a diamond). The overlaid points show the value at each observation ($n=10$ for all ROIs except transverse occipital sulcus (TOS, $n=8$) and retrosplenial cortex (RSC, $n=9$)).

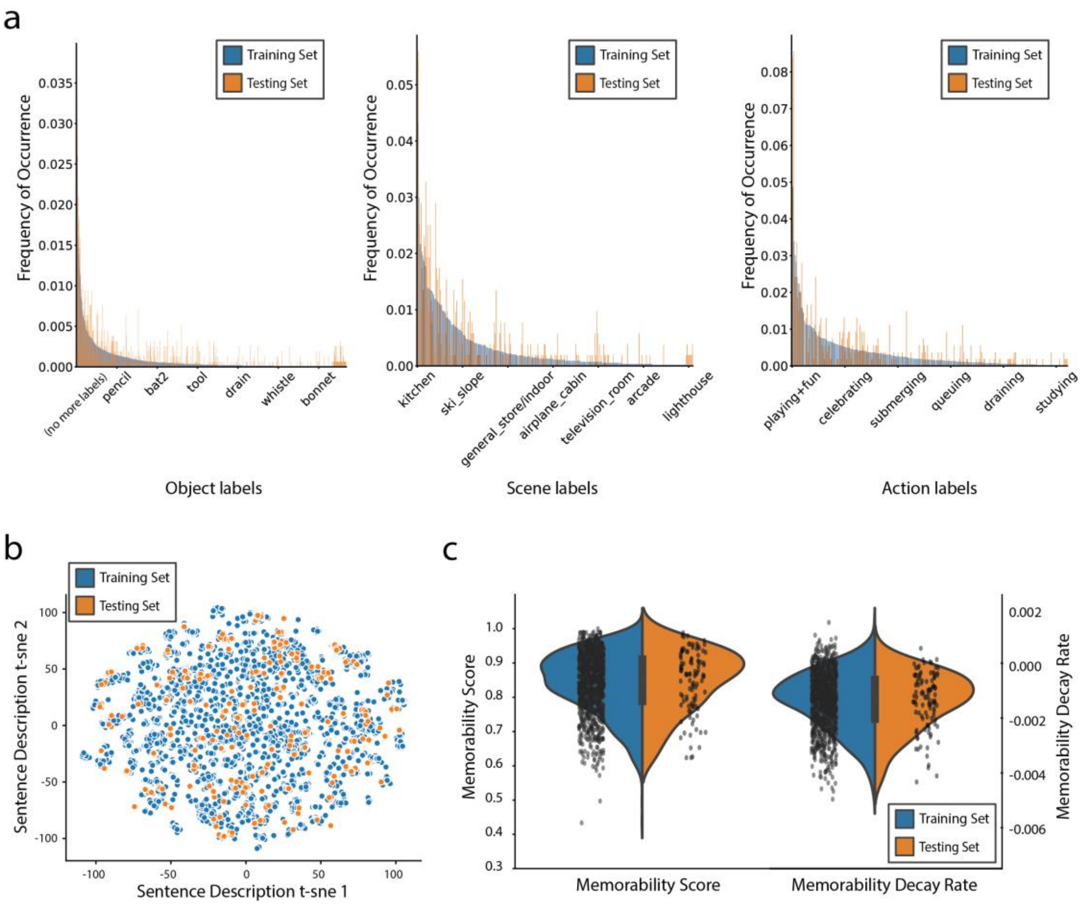

Supplementary Figure 8. **Distributions of stimuli metadata between training and testing sets.** The training and testing sets consist of 1,000 and 102 different videos, respectively. An author manually inspected the pairs of testing set videos to ensure no high-level semantic overlap, in terms of objects and actions. **a** Object, scene, and action label frequency of occurrence: The bar plot depicts the frequency of occurrence, (between 0 and 1) of, from left to right, the single-word object, scene, and action labels of the 1,102 video stimuli used in the BOLD Moments Dataset. The frequency bars for each label are separated by training (blue) and testing (orange) splits to show their similar frequency of distributions. Source data are provided as a Source Data file. **b** Text description and spoken transcription t-sne distances: The scatterplot shows the t-sne components (n=2 components, perplexity=10, number of iterations=1000) of each text description or spoken transcription embedding. The 6 sentence descriptions per video (5 text descriptions and 1 spoken transcription) serve as a useful proxy for the video's content. The t-sne plot shows the training and testing set stimuli cover similar spaces of video content. Source data are provided as a Source Data file. **c** Memorability distribution: The distribution of the memorability scores and memorability decay rates (1 per video) between the training and testing splits are highly similar and approximately normal. Source data are provided as a Source Data file. Note that the positive memorability decay rates, while theoretically implausible, reflect the true experimental results detailed in the Memento10k dataset. Users may want to set positive values to 0 depending on the analysis.

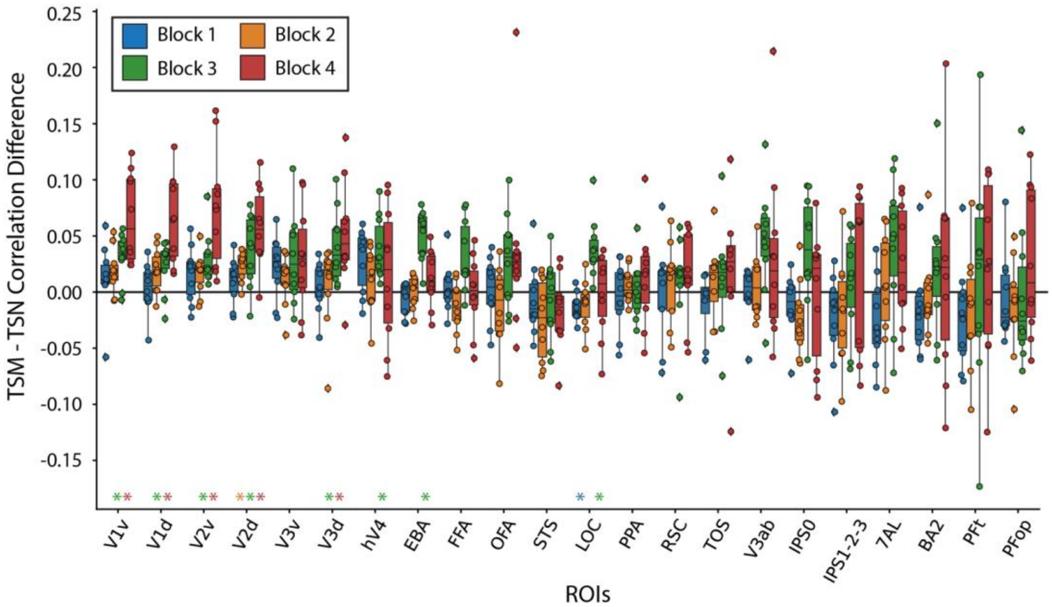

Supplementary Figure 9. **The effect of Temporal Shift Module (TSM) on brain prediction performance**. The difference in subject brain prediction performance of a Temporal Shift Module (TSM) ResNet50 and Temporal Segment Network (TSN) ResNet50 each trained on a 10,000-video subset of the M4 dataset (Multi-moments Minus Memento) was computed at each of the four Blocks for each ROI. TSM results in increased brain prediction performance most prominently in early visual ROIs. Colored asterisks along the x-axis indicates significant difference between the TSM and TSN prediction accuracy at that DNN block ($p<0.05$, one sample two-sided t-test against a population mean of 0, FDR correction across 22 ROIs x 4 blocks=88 comparisons). Source data are provided as a Source Data file. The box plot encompasses the first and third data quartiles and the median (horizontal line). The whiskers extend to the minimum and maximum values within 1.5 times the interquartile range, and values falling outside that range are considered outliers (denoted by a diamond). The overlaid points show the value at each observation (n=10 for all ROIs except transverse occipital sulcus (TOS, n=8) and retrosplenial cortex (RSC, n=9)).

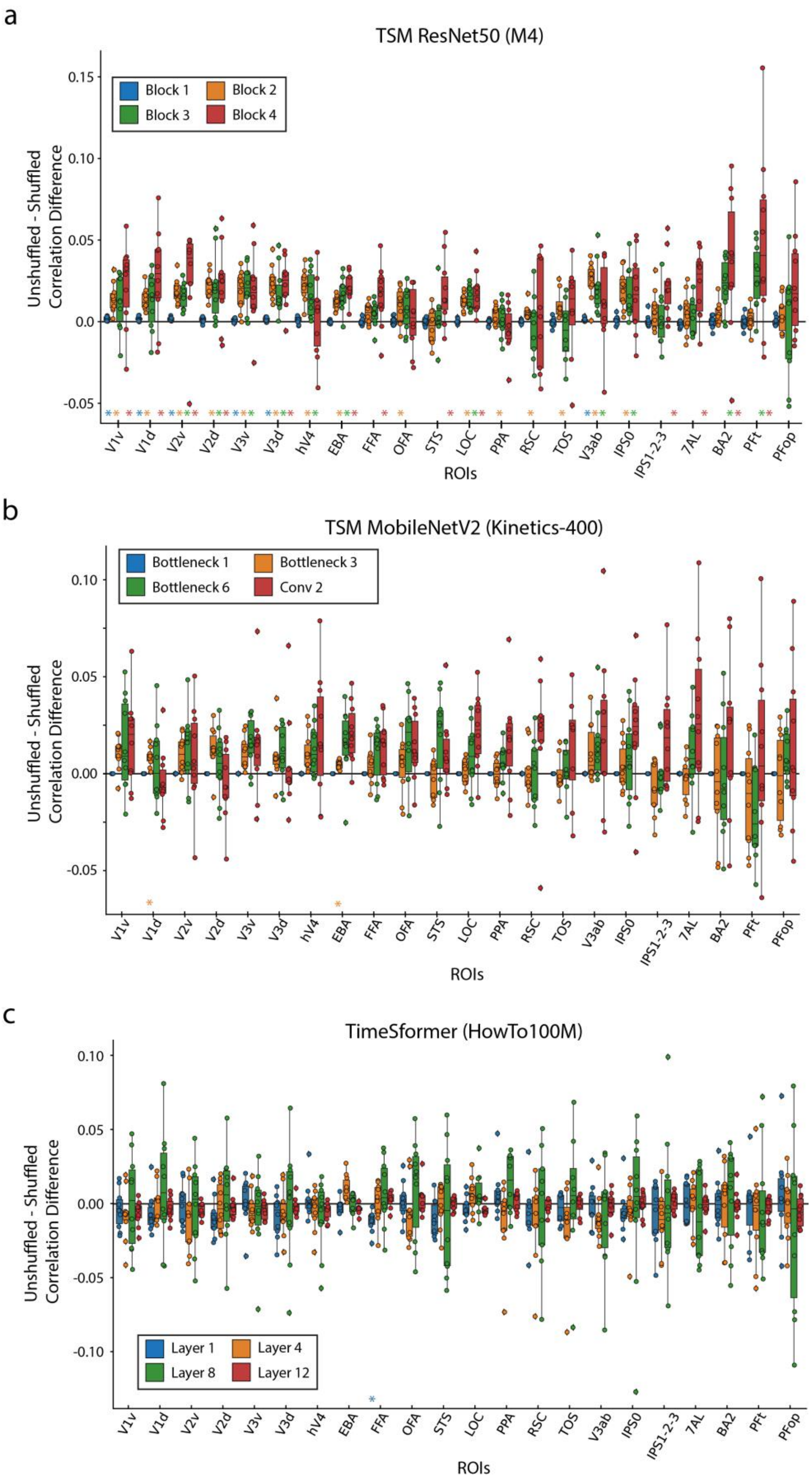
a
TSM ResNet50 (M4)
Block 1
Block 2
Block 3
Block 4
Unshuffled - Shuffled Correlation Difference
0.15
0.10
0.05
0.0
-0.05
V1v V1d V2v V2d V3v V3d hV4 EBA FFA OFA STS LOC PPA RSC TOS V3ab IPS0 IPS1-2-3 7AL BA2 PFt PFop
ROIs
b
TSM MobileNetV2 (Kinetics-400)
Bottleneck 1
Bottleneck 3
Bottleneck 6
Conv 2
Unshuffled - Shuffled Correlation Difference
0.10
0.05
0.00
-0.05
V1v V1d V2v V2d V3v V3d hV4 EBA FFA OFA STS LOC PPA RSC TOS V3ab IPS0 IPS1-2-3 7AL BA2 PFt PFop
ROIs
c
TimeSformer (HowTo100M)
Layer 1
Layer 4
Layer 8
Layer 12
Unshuffled - Shuffled Correlation Difference
0.10
0.05
0.00
-0.05
-0.10
V1v V1d V2v V2d V3v V3d hV4 EBA FFA OFA STS LOC PPA RSC TOS V3ab IPS0 IPS1-2-3 7AL BA2 PFt PFop
ROIs

Supplementary Figure 10. **The effect of frame shuffling on brain prediction performance across different architectures.** We compute the difference in the correlation between the shuffled frame prediction accuracy and unshuffled frame prediction accuracy at all 22 ROIs and four layers for **a** TSM ResNet50, **b** TSM MobileNetV2, and **c** TimeSformer model. Features were extracted at increasing levels of depth in each model (blue, orange, green, red) that reflect higher levels of model processing stages. Only the TSM ResNet50 architecture trained on the M4 dataset (Multi-moments Minus Memento10k) showed evidence of robust differences across cortex between shuffled and unshuffled input. Colored asterisks along the x-axis plot indicates significant difference between the unshuffled and shuffled prediction accuracy at that DNN block ($p<0.05$, one sample two-sided t-test against a population mean of 0, FDR correction across 22 ROIs x 4 blocks=88 comparisons). Source data are provided as a Source Data file. The box plot encompasses the first and third data quartiles and the median (horizontal line). The whiskers extend to the minimum and maximum values within 1.5 times the interquartile range, and values falling outside that range are considered outliers (denoted by a diamond). The overlaid points show the value at each observation (n=10 for all ROIs except transverse occipital sulcus (TOS, n=8) and retrosplenial cortex (RSC, n=9)).

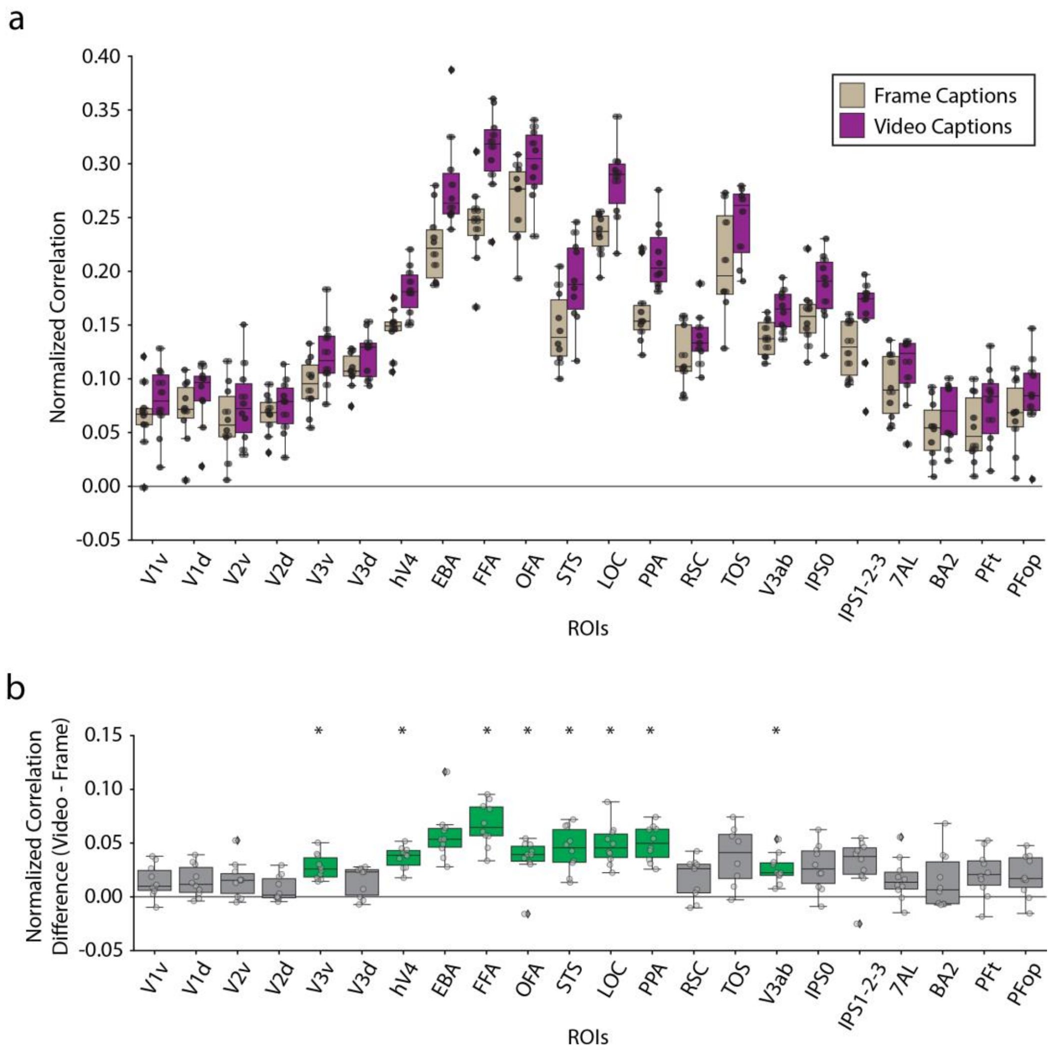

Supplementary Figure 11. **Representational similarity of frame and video captions to fMRI responses. a** ROI-based correlation: We correlate (Spearman's R) a representational dissimilarity matrix (RDM) derived from captions of short videos (purple) and captions of the middle frame of each video (beige) with an RDM at each voxel in the brain. The correlation is normalized by the voxel's upper noise ceiling. Noise-normalized correlations are averaged within each ROI and plotted for each individual subject. Five frame captions were computed from the image captioning GIT model version git-large-coco. The five video captions were human annotated and described in the Methods section Metadata subsection Text descriptions. Source data are provided as Source Data files. **b** Difference in correlations: The difference between the video-fMRI normalized correlation and frame-fMRI normalized correlation for each subject was computed and plotted. Statistically significant ROIs are colored in green and marked with a black asterisk above ($p<0.05$, one sample two-sided t-test against a null correlation of 0, Bonferroni corrected with $n=22$ ROIs). Source data are provided as Source Data files. The box plots in both panels encompass the first and third data quartiles and the median (horizontal line). The whiskers extend to the minimum and maximum values within 1.5 times the interquartile range, and values falling outside that range are considered outliers (denoted by a diamond). The overlaid points show the value at each observation ($n=10$ for all ROIs except transverse occipital sulcus (TOS, $n=8$) and retrosplenial cortex (RSC, $n=9$)).

Original Citation	Dataset Name	Number of Subjects	Number of Unique stimuli (Shared across all subjects)	Stimulus Repetitions Within Subject (Stimuli x repetitions)	Provided Stimulus Metadata?	Stimulus Superset	MRI Scanner Strength of Main Task	Auxiliary Measurements (in addition to structural)?	Other Neuroimaging Modalities	Experiment Superset
Ours	BOLD Moments (BMD)	10	1,102 3 second videos (1102)	1,000 videos x 3<br>102 videos x 10	Yes	Moments in Time<br>Multi-Moments in Time<br>Memento10k	3T	Yes	EEG (in progress)	None
1	BOLD5000	4	4,916 images (113)[a]	4,916 images x 1	Yes	SUN<br>COCO<br>ImageNet	3T	Yes	None	None
2	Forrest Gump	15	1 2-hour movie (1)	1 movie x 1	Yes	None	3T	Yes	fMRI (audio-only)[c]<br>fMRI (music listening)	StudyForrest
3	NSD	8	70,566 images (515)	10,000[d] images x 3	Yes	COCO	7T	Yes	None	None
4	Doctor Who	1	30 45-minute TV episodes (n/a)<br>7 1-3 minute video clips (n/a)	30 episodes x 1<br>7 video clips x 22	No	None	3T	Yes	None[e]	None
5	Naturalistic Neuroimaging Database (NNDb)	86	10 movies (10)[f]	1 movie x 1	No	None	1.5T	Yes	None[g]	None
6	THINGS-data	3	8,740 images (8,740)	8640 x 1<br>100 x 12	Yes	THINGS	3T	Yes	MEG<br>EEG	THINGS initiative
7	Vim-2	3	2 continuous video streams (2)	1 stream (7200 seconds) x 1<br>1 stream (540 seconds) x 10	No	None	4T	No	None	None
8	n/a	3	2 continuous video streams (2)	1 374-clip stream (2.4 hours) x 2<br>1 598-clip stream (40 minute) x 10	No	None	3T	No	None	None
9	Human Action Dataset (HAD)	30	21,600 2 second video clips (0)	720 videos x 1	Yes	Human Action Clips and Segments (HACS) Clips	3T	No	MEG (in progress)	None
10	Friends s01 - s06	6	146 22-minute TV episodes (146)[h]	1 episode x 1	No	None	3T	Yes	None	Courtois NeuroMod
10	movie10	6	4 ~1-3 hour movies (4)	2 movies x 1<br>2 movies x 2	No	None	3T	Yes	None	Courtois NeuroMod
11	Generic Object Decoding (GOD)	5	1,250 images (1,250)	1200 images x 1<br>50 images x 35	Yes	ImageNet	3T	Yes	fMRI (mental imagery)	None

[a] Chang et al., 2019: 4 subjects saw 112 images, 3 subjects saw 1 image

[b] Chang et al., 2019: Images were sampled from SUN (1000), COCO (2000), and ImageNet (1916)

[c] Hanke et al., 2016: Audio-visual fMRI was originally acquired

[d] Allen et al., 2022: Subjects each saw between 9-10,000 images

[e] Seeliger et al., 2019: Audio-visual fMRI was originally acquired

[f] Aliko et al., 2020: 8 movies were seen by 6 subjects, 1 movie was seen by 18 subjects, and 1 movie was seen by 20 subjects

[g] Aliko et al., 2020: Audio-visual fMRI was originally acquired

[h] Boyle et al., 2020: Subject 04 completed seasons 1-4 and part of season 5

Supplementary Table 1. **BMD relative to other publicly available large fMRI datasets.** We compare BMD with other large, naturalistic, task-based, visual fMRI datasets on different measures of interest (to computational neuroscientists). These measures highlight the kind of visual stimuli (type, number, overlap with existing stimuli sets, and annotations), fMRI acquisition (scanner strength, auxiliary measurements, complementary neuroimaging modalities, and subset in greater neuroimaging efforts), and experimental design (number of subjects and stimulus repetitions). This table showcases the niche each dataset fills and must not be mistaken for comparison between dataset quality or usefulness. Values may differ slightly from the original publication to facilitate comparisons across datasets and summarize information. Note that while some datasets are not officially part of a larger experiment superset, many have been used in independent studies and thus may have additional stimuli metadata and neuroimaging data. Such cases are not noted in this table to maintain clarity. Please see the original publication for the most accurate information.

The added value of a short video versus a static image neuroimaging dataset

We emphasize that a short video (e.g., 3 second duration, as in BMD) fMRI dataset is not better or worse than a static image fMRI dataset; rather, they are different in terms of stimulus features and corresponding brain responses that may make one better suited to answer specific research questions. Most obvious, short videos contain a naturalistic temporal dimension that static images do not, allowing the video to communicate crucial contextual information about how spatial components in our environment move (or not) and spatially relate to each other over time. The benefit of this temporal dimension is clear in our everyday lives – we can interpret transitions between states (a door is being opened, not closed), direction (a steering wheel is being turned to left, not right or still), reactions (the child laughed after being shown the picture), motion (the baby is crawling slowly, not fast), and more.

The contextual value of a video's temporal dimension is reflected in BMD's own action and sentence text description metadata. Concerning action labels, images can only be labelled with a limited subset of actions or be highly constrained to capture a specific action. For example, the action of a baseball player "hitting" the ball can only be captured with an image if the photo were taken at a very specific instant in time. Otherwise the action may be "standing" or "swinging". A short 3s video, as in BMD, easily captures these actions without heavily constraining the space of possible videos that correspond to "hitting". Concerning text descriptions, short videos can capture temporal sequences of events that an image cannot. We contrast these video captions with captions of only each video's middle frame (frame captions generated by GIT [12] below (emphasis our own):

Video 0001:

- Video caption: "A mallard is in the water alone *swimming around* and *putting its beak in.*"
- Frame caption: "A duck floating on top of a blue body of water."

Video 0002:

- Video caption: "A man *is showing another man* how to *move feet back and forth*."
- Frame caption: "a couple of men standing in a garage."

Video 0005:

- Video caption: "A woman guides a little boy's arms *up and down as other kids stretch* around him."

- Frame caption: “a group of children standing around a room.”

Video 0006:

- Video caption: "a chess tournament is going on this is focused on two players one *is moving their queen and taking something* to put the king in checkmate"
- Frame caption: “a group of people sitting at tables playing chess.”

Static frames of these videos cannot capture the temporal facts that the mallard is “putting its beak in”, the man “is showing another man how”, “a woman guides…as other kids stretch”, and a chess player “is moving their queen and taking something to put the king in checkmate.” This temporal information adds valuable context that often makes one’s understanding of the 3s video vastly richer compared to any single static frame.

These differences in short videos and static images also translate to differences in fMRI brain responses. Previous work has found that videos evoke a greater extent [13–17] and pattern [18–21] of cortex responding to videos than images throughout occipito-temporal, dorsal visual, and parietal cortex. In this manuscript we describe our highly reliable activations throughout cortex (Fig. 3) with notably high reliability in parietal cortex, a region of the brain that weakly responds to static images. These highly reliable brain responses are not just a result of increased participant engagement or stimulus saliency; we even show that BMD brain responses capture temporal information from the videos (Fig. 5, Fig. 6, Supplementary Fig. 9, Supplementary Fig. 13c) despite the BOLD response’s temporal sluggishness and fMRI’s low sampling rate. We further show that the full video captions lead to higher representational similarity with BMD’s brain responses than the frame captions through much of the ventral visual cortex (Supplementary Fig. 11).

In the adjacent field of computer vision, researchers have long recognized that videos and images demand different modeling approaches [22–26] and training datasets [27–31] for strong task performance. Videos continue to be at the forefront of ground breaking computer vision research due to their creative, cross-domain, and practical applications in text-to-video generation [32–34], video understanding with large language models [35–37], and efficient action recognition and pose estimation [38–40].

Taken together, short video fMRI datasets offer unique opportunities to advance computational neuroscience where static image fMRI datasets do not. They can advance methodologies around estimating BOLD signals in response to rapid stimulus presentations [41–43], elucidate cognitive functions concerning temporal integration [44–46], test temporally specific cognitive objective functions [47,48], and detail how multiple visual pathways interact to achieve an understanding of an event [20,21,49,50]. As neuro and computer science research become increasingly intertwined [3,51–53], BMD is well-suited to integrate with state-of-the-art video modeling work from the computer vision community. Importantly, a short video dataset like BMD can make these scientific advancements while staying connected to the vast body of still image work by sharing event-related paradigms, multivariate and univariate methodologies, representational similarity analyses, and/or encoding and decoding techniques. Short video datasets offer more ecological validity than static images while retaining experimental control and offer tremendous potential to advance our understanding of the human visual system.

Structural and functional scan quality assessment

We use MRIQC [54] to measure the quality of our study's original or minimally preprocessed structural and functional MRI scans. MRIQC is an open-source software that outputs a large and diverse set of image quality metrics (IQMs) to comprehensively quantify the quality of (f)MRI data in a standardized and reproducible manner. IQMs are calculated at the level of a single run, and group reports are generated for all T1w, T2w, and BOLD runs in the study. We present a representative subset of 6 IQMs to summarize the quality of our structural scans and another subset of 6 IQMs to summarize the quality of our functional scans (see MRIQC documentation for details on all 112 IQMs: https://mriqc.readthedocs.io/en/latest/measures.html). Note that no set of metrics can fully describe data quality by itself. Thus, when choosing IQMs to represent the structural and functional scan quality, we considered the following three criteria:

First, the representative IQMs for the structural scans and for the functional scans should capture metrics especially relevant to the properties of structural and functional scans.

Second, IQMs that are useful for describing the quality of both structural and functional scans are preferred in order to create more cohesive and shared IQM subsets between the structural and functional scans.

Third, IQMs commonly reported in previous literature are preferred in order to improve comparisons across studies and be more familiar to readers.

We additionally use MRIQCeption to contextualize our study's group reported results within a large collection of anonymized group reports from studies of comparable scanner parameters (1 < Tesla < 3, 1 <= TR < 3).

For structural (T1w and T2w) scans, we present the results from the following IQMs:

SNR Total - Signal to Noise Ratio: SNR Total for structural scans is computed by averaging the SNR across the cerebrospinal fluid (snr_csf), gray matter (snr_gm), and white matter (snr_wm). SNR is calculated by the following formula:

$$SNR\ Total = \frac{\mu_F}{\sigma_F\sqrt{n(n-1)}} \tag{3}$$

Where μ_F is the mean intensity of the foreground, σ_F is the standard deviation of the foreground intensity, and n is the number of voxels in the foreground mask. Higher values correspond to higher quality.

CNR - Contrast to Noise Ratio: CNR, an extension of SNR, computes the absolute value difference of the gray and white matter image values ($|S_W - S_G|$) and divides them by the standard deviation of the values in the surrounding air (σ_{air}). Higher values correspond to higher quality.

CJV - Coefficient of Joint Variation: CJV is the ratio of the coefficient of variation in the gray matter to the coefficient of variation in the white matter. Lower values correspond to higher quality.

EFC - Entropy Focus Criterion: EFC is the Shannon entropy of voxel intensities normalized by the maximum Shannon entropy value. It measures ghosting and blurring due to head motion. Lower values correspond to higher quality.

FWHM Avg - Average Full-Width Half Maximum Smoothness: FWHM Avg is the average spatial distribution of voxel intensities in an image using a Gaussian width estimator. Lower values correspond to higher quality.

FBER - Foreground-Background Energy Ratio: FBER is the ratio of the mean energy inside the head to the mean energy outside the head. Higher values correspond to higher quality.

For functional scans, we present the results from the following IQMs:

SNR - Signal to Noise Ratio: SNR for functional scans is calculated by the following formula:

$$SNR = \frac{\mu_F}{\sigma_F\sqrt{n(n-1)}} \tag{4}$$

Where μ_F is the mean intensity of the foreground, σ_F is the standard deviation of the foreground intensity, and n is the number of voxels in the foreground mask. Higher values correspond to higher quality.

tSNR - Temporal Signal to Noise Ratio: tSNR divides the mean BOLD signal across time by the temporal standard deviation map. Higher values correspond to higher quality.

FD Mean - Mean Framewise Displacement: FD Mean computes the average displacement of all six motion parameters. Lower values correspond to higher quality.

FWHM Avg - Average Full-Width Half Maximum Smoothness: FWHM Avg is the average spatial distribution of voxel intensities in an image using a gaussian width estimator. Lower values correspond to higher quality.

AOR - AFNI Outlier Ratio: AOR is the average fraction of outliers found in each fMRI volume as computed by AFNI's "3dToutcount" function. Lower values correspond to higher quality.

AQI - AFNI Quality Index: AQI computes the average distance between each volume and the median volume of a series, given by AFNI's "3dTqual" function. Lower values correspond to higher quality.

The Algonauts Project 2021 challenge approaches of the top three winners

The Algonauts Project 2021: How the Human Brain Makes Sense of a World in Motion is an open challenge that took place during the spring and summer of 2021 and culminated in an interactive workshop and speaking event at the Computational Cognitive Neuroscience (CCN) conference [53,55]. For the challenge, participants submit the predictions of their computational model on held-out brain data (see http://algonauts.csail.mit.edu/challenge.html for the final challenge leaderboard and details). We summarize the top three challenge entries, highlighting their different modeling approaches and insights at the intersection of natural and artificial intelligence research.

The first-place team “huze” approached this challenge using an ensemble of 6 different models that together integrate meaningful features of video understanding: spatiotemporal, motion, edge, and audio features [56]. They then weighted the outputs of each model representation and found that the predictivity for each ROI was highest when combining features from all models. They additionally optimized the receptive field size for each of the four I3D RGB model layers and ROI [30]. They showed that early ROIs benefited most from smaller receptive fields on low-level layers (layers 1 and 2) and later ROIs benefited most from larger receptive fields on high-level layers (layers 3 and 4), replicating neuroscience results [57].

The second-place team “bionn” was interested in evaluating a range of DNNs from the more classical supervised CNNs (AlexNet, VGG19, ResNet50, and ResNet152) to the more modern contrastive learning and visual transformer networks (simclr, pclv2, and visual transformer network ViT) [58]. They found the ResNet models, specifically ResNet152, outperformed the visual transformer and contrastive learning networks. Similar to “huze”, “bionn” also took advantage of pooling the model features to simulate small receptive fields for early regions and large receptive fields for later regions.

The third-place team “shinji” [59] experimented with state-of-the-art spatiotemporal vision features from TimeSformer [23] and classical, neurophysiology-based motion energy features [7,60]. Looking exclusively at the TimeSformer model, they first saw that earlier layers (layers 4-6 out of 12) best predicted early visual regions (V1-V4) while later layers (layers 9-11 out of 12) best predicted later visual regions (EBA, LOC, STS, FFA, and PPA). In early visual regions (V1-V3), the motion-energy model outperformed the TimeSformer model, and in the later visual regions (V4, EBA, LOC, STS, FFA, and PPA), the

TimeSformer model was better. However, the combination of both the TimeSformer and motion-energy features was best for all ROIs except for FFA, STS, and PPA.

For more details about the approaches of the top three challenge winners, see the PDFs of their full reports, available online or with the BMD dataset.

Version B preprocessing pipeline

Overview

Here we detail an additional preprocessed version (version B) of the BOLD Moments Dataset (BMD) released alongside the version presented in the manuscript (version A). Version B offers additional flexibility to use BMD in a researcher's desired output space and ROI format (see Supplementary Fig. 12). In brief, version B is preprocessed in five output spaces (MNI152NLin2009cAsym, anatomical, fsaverage, fsnative, fsLR32k), contains beta estimates computed with GLMsingle [42] for MNI152NLin2009cAsym, fsLR32k, and fsaverage spaces, and defines 47 ROIs in MNI152NLin2009cAsym space (Supplementary Fig. 13b, left and right hemispheres of the 22 ROIs defined in the main text and MT, plus one "BMDgeneral" ROI). We show whole brain noise ceiling reliability results in the volume-based MNI152NLin2009cAsym (Supplementary Fig. 13a) and surface-based fsLR32k (Supplementary Fig. 14) spaces and high predictivity of a motion energy model in motion-selective ROIs (MT, hV4, V3AB, IPS0) (Supplementary Fig. 13c).

Both version A (presented in the manuscript) and version B (described here) are identical up to fMRIPrep [61] preprocessing (see Supplementary Fig. 12). Details on the experimental design, participants, and MRI acquisition protocols can be found in the main text. Version A was preprocessed using the default 6 degrees of freedom for BOLD to T1w image registration (the flag –bold2t1w-dof) and one standard volumetric output space (MNI152NLin2009cAsym). Version B was preprocessed with 12 degrees of freedom for BOLD to T1w image registration and five output spaces comprising a standard and native volume output (MNI152NLin2009cAsym, anat) and two standard and native surface outputs (fsaverage, fsLR32k, fsnative) with transformation matrices available between the spaces (see fMRIPrep preprocessing boilerplate text below). Registration to the fsLR32k space takes advantage of scripts for the minimal preprocessing pipeline used in the Human Connectome Project [62,63]. This registration uses the CIFTI format, where cortical structures are organized in 2D surface-based "grayordinates" and subcortical structures are organized in 3D volume-based voxels. This registration provides excellent volume-to-surface registration, especially for inter-subject analyses, and access to a suite of HCP analysis and visualization tools [62,64].

We provide single trial beta estimates using GLMsingle [42] in the volume-based MNI152NLin2009cAsym and surface-based fsLR32k and fsaverage output spaces. We also define 47 ROIs in the MNI152NLin2009cAsym volume space for greater research and modeling flexibility. The 47 ROIs are similar to the 22 ROIs described in the main manuscript but are separated by left and right hemisphere,

include the motion-selective MT ROI, include a “BMDgeneral” ROI that broadly defines reliably activated cortex across all BMD subjects, and enforce ROIs to have an equal number of voxels across subjects to facilitate inter-subject modeling.

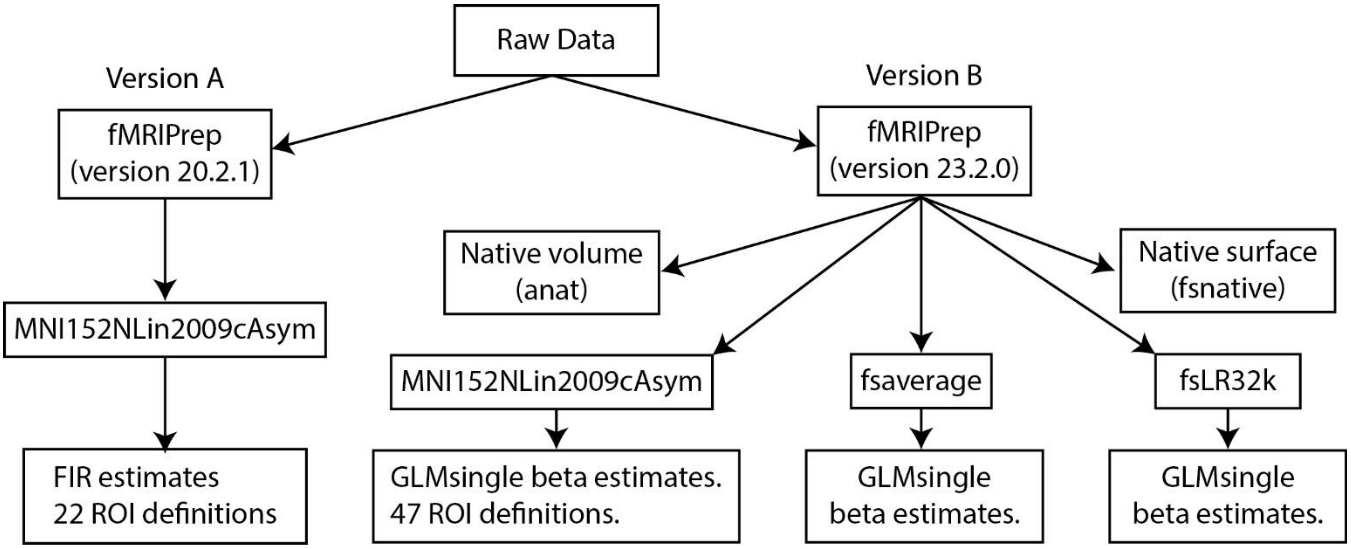

Supplementary Figure 12. **Overview of preprocessing pipelines.** Version A (left) of BMD was preprocessed with fMRIPrep into a standard volumetric output space, modeled with FIR functions, and supplemented with 22 ROI definitions. Details are provided in the main manuscript. Version B (right) of BMD was preprocessed with fMRIPrep into two volume-based and three surface-based output spaces. Single trial beta estimates using GLMsingle and 47 ROI definitions were provided in the standard volume-based space.

Version B MNI152NLin2009cAsym, fsLR32k, and fsaverage preprocessing

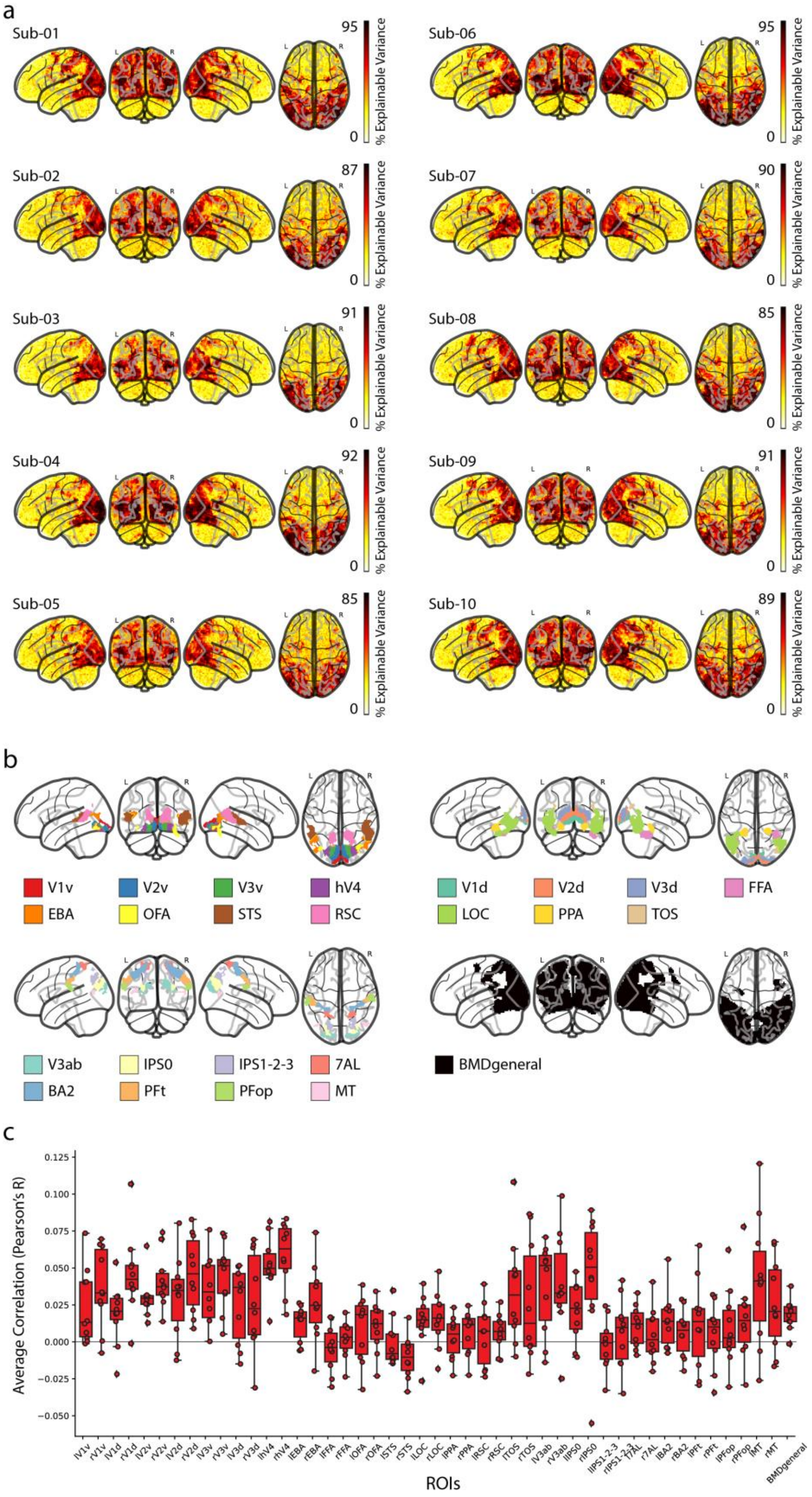

Supplementary Figure 13. **Whole-brain noise ceiling and regions of interest (ROIs). a** For each subject and voxel in the whole brain, we show the noise ceiling as percent of explainable variance using the testing set videos. **b** We show the 46 non-overlapping parcels (combined left and right hemispheres) and BMDgeneral (black) in a glass brain. Each subject's 8 category-selective ROIs (EBA, OFA, STS, RSC, FFA, LOC, PPA, and TOS) are functionally defined by extracting the top 50% most active voxels within the respective parcel. All subjects share the same ROI definition from the remaining parcels. BMDgeneral is defined independently from the ROIs and reflects a group-averaged region of cortex that reliably responds to videos in the BOLD Moments experiment. **c** The boxplots depict the correlation (Pearson) between the predicted brain responses with the true responses of the testing set using stimulus features computed from a motion energy model. The boxes show the median response across subjects (horizontal line), 25th and 75th percentile (lower and upper box boundary), and whiskers extending to maximum and minimum values within 1.5 times the interquartile range. Individual subject results are shown as black points, and outliers are shown as diamonds (n=10 subjects for all ROIs).

All fMRI data were organized in the standardized BIDS format [65] and preprocessed using fMRIPrep [61]. The data were slice time corrected to 0s, co-registered to the subject's T1w anatomical scan, and registered to standard and nonstandard output spaces (MNI152NLin2009cAsym, fsLR32k, fsaverage, fsnative, native volume). Registration to fsLR32k space employs the MSMSulc algorithm [64] for more accurate alignment of the cortical surface by weighting voxels along the cortical ribbon and projecting to vertices on the surface (e.g., “grayordinates”). Subcortical voxels (including cerebellum) are resampled to volumetric MNI space.

The main experimental runs were then temporally interpolated from their acquisition TR of 1.75 seconds to a TR of 1 second to time-lock volume sampling to stimulus presentations. A General Linear Model (GLM) was used to estimate single trial beta estimates [42] in the MNI152NLin2009cAsym, fsLR32k, and fsaverage spaces. The beta responses were then normalized (z-scored) within each scanning session across conditions.

Note that preprocessing BMD through the entire HCP preprocessing pipeline is expected to obtain even better results in fsLR32k due to BMD’s availability of high resolution T2w and fieldmap scans. We use the fsLR32k registration native to the fMRIPrep preprocessing tool to maintain a common root between output spaces all while still making the data immediately available in the advantageous CIFTI format.

General linear model

Functional localizer

We use GLMsingle [42] to model the fMRI response to the video localizer for each subject separately. The subject’s preprocessed data was spatially smoothed with a 9mm full width half maximum of the

Gaussian kernel. The data was then temporally interpolated from an acquisition TR of 1.75s to an interpolated TR of 1s to timelock image acquisition to block onset. Each block, although composed of 6 3s videos (except for the fixation blocks, where no videos were shown), was modeled as a single stimulus. The onsets and durations (18s) of the Body, Face, Object, Scene, and Scrambled blocks, along with the temporally interpolated and smoothed fMRI time series, were input to the general linear model. GLMsingle (1) chose an optimal HRF from a library at each voxel, (2) identified a number of nuisance regressors from principal component analysis of a noise pool that explain a maximum amount of variance, and (3) performed fractional ridge regression at each voxel to estimate single trial betas.

Main experiment

For each subject, we fit beta estimates to each single-trial fMRI response in the main experiment using GLMsingle [42]. The preprocessed data was temporally interpolated from an acquisition TR of 1.75s to an interpolated TR of 1s to time-lock stimulus onset to image acquisition (1.75s does not evenly divide the inter-trial interval of 4s). In this way, we acquire fMRI scans at different timepoints along the BOLD signal (with respect to stimulus presentation) and, after interpolating, achieve a regular sampling of the BOLD signal time-locked to stimulus onset for easier analysis. The interpolated fMRI time series, stimulus onsets, and stimulus durations (modeled with 3s durations) for each session separately were input to the general linear model. GLMsingle estimated single trial beta values by (1) fitting an optimal HRF to each voxel from a library of HRFs, (2) identifying nuisance regressors from a noise pool that maximally explain variance, and (3) implementing fractional ridge regression to improve estimates in a rapid event-related design. Responses for both the training and testing videos within a session were estimated with the same GLM to take advantage of the testing set's multiple repetitions for GLMsingle's type-d estimations.

In this way, we obtained a single beta estimate for each stimulus presentation for each subject. This resulted in a total of 4,020 beta estimates per subject (3 beta estimates x 1,000 training videos and 10 beta estimates x 102 testing videos).

The beta estimates were normalized within each scanning using the session's training set mean and standard deviation. Specifically, the mean and standard deviation at each voxel across the session's training set videos were computed. The mean was subtracted from the training set estimates and the testing set estimates, and the standard deviation was divided from the data. For the data in MNI152NLin2009cAsym space, nan-indices corresponding to outside the subject's brain mask were identified and removed.

MNI152NLin2009cAsym Regions of interest definition

We computed a non-overlapping set of 46 ROIs (regions of interest) (23 ROIs separated by left and right hemispheres) previously known to be driven by dynamic stimuli spanning visual and parietal cortices [66–73] (Supplementary Fig. 13b). Note that these ROI definitions differ slightly compared to those detailed in the main manuscript (version A). We first created a non-overlapping parcellation in the standard MNI152NLin2009cAsym space identical across subjects composed of parcels resampled from Wang and colleagues, Glasser and colleagues, and Julian and colleagues [63,74,75]. Finally, we used the t-contrasts from each subject's functional localizer results to identify the top 50% of voxels within the corresponding functional parcel from Julian and colleagues [74] (bodies > objects: EBA; objects > scrambled: LOC; scenes > objects: PPA, RSC, STS; faces > objects: OFA, FFA, STS). This ROI definition method facilitated inter-subject modeling approaches by ensuring all ROIs were defined for each subject and each ROI contained the same number of voxels across subjects. Furthermore, the parcellation shared across subjects (before taking each subject's top 50% of voxels in the parcels from Julian and colleagues [74]) allowed modeling approaches that incorporate voxel-level spatial information, since the parcel indices are the same for each subject.

In detail, we defined ROIs V1v, V1d, V2v, V2d, V3v, V3d, hV4, V3a, V3b, IPS0, IPS1, IPS2, IPS3 from Wang and colleagues [75] (maxprob_vol_{h}h.nii, where {h} is "l" or "r"), 2 (here referred to as BA2), 7AL, PFt, PFop, and MT from Glasser and colleagues [63] (MNI_Glasser_HCP_2019_v1.0.nii available from afni.nimh.nih.gov), and EBA, LOC, PPA, RSC, STS, OFA, FFA, and STS from Julian and colleagues [74] ({h}{parcel}.img from the n=30 group, where {h} is "l" or "r" and {parcel} is the parcel name). We group V3a and V3b into V3ab and IPS1, IPS2, and IPS3 into IPS1-2-3 due to subtle differences in functional preferences that can be difficult to resolve with our in-the-wild naturalistic stimuli [14,15,76,77]. All ROIs were resampled into our functional volumetric dimensions and separated by left and right hemispheres. Voxels outside a common brain mask computed across subjects were removed from the parcel. There was no overlap between the parcels within Wang and colleagues [75] or between the parcels within Glasser and colleagues [63].

To address minimal overlap between the parcels derived from Julian and colleagues [74], the in-question voxels were assigned to the parcels that had a greater inter-subject agreement from our functional localizer experiment. Specifically, a t-test (two-sided, independent) was computed between the condition beta estimates to define category-selective contrasts: bodies > objects (body selective; EBA), objects > scrambled (object selective; LOC), scenes > objects (scene selective; PPA, RSC, TOS), and faces > objects (face selective; OFA, FFA, STS). Similar to the Group-constrained Subject Specific

(GSS) procedure [74], the t-contrast maps were binarized at a p-value cutoff ($p < 0.05$, uncorrected), where voxels below this cutoff were assigned 0 and voxels above this cutoff were assigned 1. The binarized t-contrast maps were averaged across subjects and smoothed (6mm full width half maximum of the Gaussian kernel) to obtain four probability maps (one for each contrast) that contains information on inter-subject agreement at each voxel. If both LOC and EBA overlapped at voxel A, for example, we indexed voxel A's value in both the objects > scrambled probability map (for LOC) and the bodies > objects probability map (for EBA) and assigned voxel A to the parcel with the higher value (i.e., the higher inter-subject agreement). No parcels within a contrast overlapped.

Finally, we addressed the minimal overlap between parcels across the three atlases. If non-functionally defined parcels (i.e., the parcels derived from Wang and colleagues [75] and Glasser and colleagues [63]) overlapped with functionally-defined parcels (i.e., from Julian and colleagues [74]), preference was given first to the non-functionally defined parcel. Otherwise, the voxel may end up not being assigned to any ROI after subject-specific ROI definition (described below) because the functionally-defined parcels are generous in size, reflecting group-level inter-subject agreement. If the overlapping parcels were all non-functionally defined, preference would have gone to the smaller parcel to preserve its size, but there was only overlap between functionally and non-functionally defined parcels. In this way, we obtained 46 non-overlapping parcels identical for each subject.

We then functionally define the category-selective ROIs for each subject by identifying the top 50% most active (i.e., highest t-values, uncorrected) voxels inside the ROIs respective t-contrast map masked by the corresponding parcel [78]. This method achieves both a subject-specific functional definition, maintains the relative size between ROIs, and ensures the same number of voxels within an ROI across subjects. No constraint of contiguity was enforced.

We additionally define a swath of cortex that showed consistently reliable responses to videos across subjects in this study, here termed BMDgeneral (Supplementary Fig. 13b). BMDgeneral was algorithmically defined in five steps: (1) compute split-half correlations between two randomly selected repetitions of all 1,102 stimuli repetitions to obtain p-values at each voxel (Pearson R correlation, two-sided, p-values averaged over 10 sets of split-half correlations), (2) binarize the volume at $p < 0.05$ (uncorrected), indicating voxels with a value of 0 and 1 have poor and good split-half reliability, (3) average each subject's binarized mask to obtain a probability map reflecting the inter-subject agreement of each voxel's reliability, (4) smooth the probability map (6mm at full width half maximum), and (5) identify clusters (cluster threshold = 50 voxels, 8mm between peaks, statistic threshold of 0.1).

All identified clusters are collectively identified as BMDgeneral. BMDgeneral may or may not overlap with the 46 ROIs.

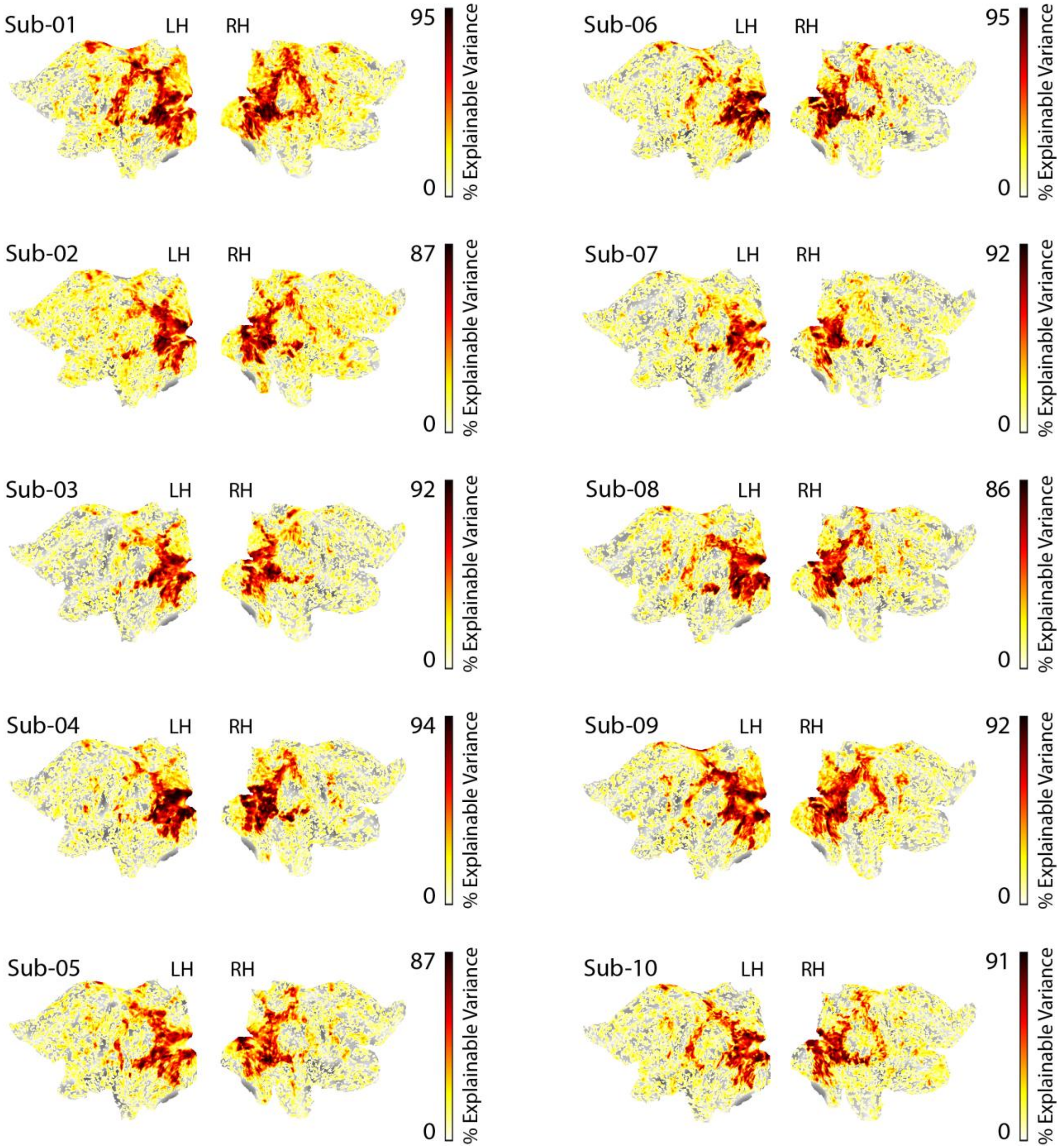

Supplementary Figure 14. **Left and right cortex noise ceiling.** For each subject and grayordinate vertex in the left and right flattened hemispheres, we show the noise ceiling as percent of explainable variance using the testing set videos. Values are thresholded at 1.

Noise ceiling calculation

We compute the percent of explainable variance at each voxel for each subject as an estimate of the noise ceiling (equation 6) [3,42]. First, the noise, signal and total variance of the beta estimates are

computed. The noise variance is computed as the mean variance of the beta estimates of the within-video presentation trials. The total variance is computed as the variance of the beta estimates across all video presentation trials. The signal variance is computed as the total variance minus the noise variance. The signal variance was positively rectified, where negative values were assigned 0 and positive values were preserved. Next, the noise ceiling signal-to-noise ratio (SNR) (*ncsnr*) is computed as the fraction of signal standard deviation (σ_{signal}) to noise standard deviation (σ_{noise}) (equation 5),

$$ncsnr = \frac{\sqrt{var_{signal}}}{\sqrt{var_{noise}}} \tag{5}$$

where the standard deviation is equal to the square root of the variance ($\sigma = \sqrt{var}$). Finally, the percentage of explainable variance (*noise ceiling*) is calculated as,

$$noiseceiling = \frac{ncsnr^2}{ncsnr^2 + {}^{1}/_{numTrials}} * 100 \tag{6}$$

where *ncsnr* is the noise ceiling signal-to-noise ratio (SNR) and *numTrials* is the number of video presentation trials (*numTrials*=10 for the testing set and *numTrials*=3 for the training set). This measure of voxel reliability differs from the split-half reliability measure used in the main manuscript (version A) but are mathematically related and produce similar results. The reliability measure proposed here is computationally less expensive than a split-half computation and has no stochastic elements. Noise ceiling estimates are shown for each subject in both the MNI152NLin2009cAsym space (Supplementary Fig. 13a) and fsLR32k space (Supplementary Fig. 14).

Motion energy features computation and encoding model

Motion energy features were used to predict brain activity in response to BMD's 3 second naturalistic videos. The motion energy model [7,60,79] consists of a series of spatial and temporal Gabor filters intended to capture local motion and direction in a video stimulus, thus making it a highly interpretable method to model video dynamics. The motion energy encoding model accuracy (Supplementary Fig. 13C) shows high prediction accuracy in motion selective ROIs, namely MT [80,81], hV4 [82,83], V3AB [14,77], and IPS0 [14]. These results support that single trial beta estimates of BMD's 3 second naturalistic videos capture motion information.

Motion energy features for each BMD video stimulus was computed using the MATLAB code available here: https://github.com/gallantlab/motion_energy_matlab [7,81]. For each 268x268 video, the frames were converted from RGB to LAB color space, and only the L (luminance) channel was retained. The luminance channel was then passed through a three-dimensional bank of spatiotemporal Gabor filters

consisting of two spatial dimensions and one temporal dimension. Similar to the filter bank used in [7] to model naturalistic movies, the three-dimensional filters are defined at five spatial frequencies (0, 2, 4, 8, 16, and 32 cycles/image), three temporal frequencies (0, 2, and 4Hz), and eight directions (0, 45, 90, 135, 180, 225, 270, and 315 degrees) with the exception that the 0 Hz temporal filter is defined at only 0, 45, 90, and 135 degrees directions and the 0 cycles/image spatial filter is defined at 0 degree orientation. Local motion-energy features were computed by taking the square root of the sum of the squared outputs of each pair of filters with orthogonal phases. The logarithm of the output from these filters was computed to scale large values, and the temporal dimension of the output was downsampled to 1 second to match the fMRI sampling rate (i.e., the interpolated TR of 1 second) of the BOLD time series. The output was then z-scored across time. In total, this procedure resulted in a matrix of size 3 x 6,555 (seconds x motion energy features).

The motion energy features were then used in a voxelwise linear encoding model [84] to predict the brain activity (beta estimates) in 47 regions of interest (ROIs) from the version B preprocessed data in MNI152NLin2009cAsym space (Supplementary Fig. 13C). Specifically, the motion energy features for each video were concatenated along the three seconds and underwent principal component analysis (PCA) to reduce dimensionality to the number of components that explained 95% of the variance. PCA was fit to the training videos and applied to both the training and testing videos. A linear model was then fit to the training video features to predict the response at the voxel. The learned weights of the linear model were then applied to the testing video features. The encoding model accuracy was computed as the correlation of the vector of predicted responses of the test set with the vector of true responses of the test set.

Version B fMRIPrep preprocessing boilerplate text

We reproduce the fMRIPrep boilerplate text describing version B's preprocessing details below (indented):

> Results included in this manuscript come from preprocessing performed using fMRIPrep 23.0.2 ([61,85]; RRID:SCR_016216), which is based on Nipype 1.8.6 ([86,87]; RRID:SCR_002502).
>
> **Preprocessing of B0 inhomogeneity mappings**
>
> A total of 6 fieldmaps were found available within the input BIDS structure for this particular subject. A B0 nonuniformity map (or fieldmap) was estimated from the phase-drift map(s) measure with two consecutive GRE (gradient-recalled echo) acquisitions. The corresponding phase-map(s) were phase-unwrapped with prelude (FSL None).

Anatomical data preprocessing

A total of 1 T1-weighted (T1w) images were found within the input BIDS dataset. The T1-weighted (T1w) image was corrected for intensity non-uniformity (INU) with N4BiasFieldCorrection [88], distributed with ANTs 2.5.0 ([89]; RRID:SCR_004757), and used as T1w-reference throughout the workflow. The T1w-reference was then skull-stripped with a Nipype implementation of the antsBrainExtraction.sh workflow (from ANTs), using OASIS30ANTs as target template. Brain tissue segmentation of cerebrospinal fluid (CSF), white-matter (WM) and gray-matter (GM) was performed on the brain-extracted T1w using fast (FSL (version unknown), RRID:SCR_002823, [90]). Brain surfaces were reconstructed using recon-all (FreeSurfer 7.3.2, RRID:SCR_001847, [91]), and the brain mask estimated previously was refined with a custom variation of the method to reconcile ANTs-derived and FreeSurfer-derived segmentations of the cortical gray-matter of Mindboggle (RRID:SCR_002438, [92]). A T2-weighted image was used to improve pial surface refinement. Brain surfaces were reconstructed using recon-all (FreeSurfer 7.3.2, RRID:SCR_001847, [91]), and the brain mask estimated previously was refined with a custom variation of the method to reconcile ANTs-derived and FreeSurfer-derived segmentations of the cortical gray-matter of Mindboggle (RRID:SCR_002438, [92]). Volume-based spatial normalization to two standard spaces (MNI152NLin2009cAsym, MNI152NLin6Asym) was performed through nonlinear registration with antsRegistration (ANTs 2.5.0), using brain-extracted versions of both T1w reference and the T1w template. The following templates were were selected for spatial normalization and accessed with TemplateFlow (23.1.0, [93]): ICBM 152 Nonlinear Asymmetrical template version 2009c [[94], RRID:SCR_008796; TemplateFlow ID: MNI152NLin2009cAsym], FSL's MNI ICBM 152 non-linear 6th Generation Asymmetric Average Brain Stereotaxic Registration Model [[95], RRID:SCR_002823; TemplateFlow ID: MNI152NLin6Asym]. Grayordinate "dscalar" files containing 91k samples were resampled onto fsLR using the Connectome Workbench ([62]).

Functional data preprocessing

For each of the 62 BOLD runs found per subject (across all tasks and sessions), the following preprocessing was performed. First, a reference volume and its skull-stripped version were generated using a custom methodology of *fMRIPrep*. Head-motion parameters with respect to the BOLD reference (transformation matrices, and six

corresponding rotation and translation parameters) are estimated before any spatiotemporal filtering using mcflirt (FSL 6.0.5.1:57b01774, [96]). The estimated fieldmap was then aligned with rigid-registration to the target EPI (echo-planar imaging) reference run. The field coefficients were mapped on to the reference EPI using the transform. The BOLD reference was then co-registered to the T1w reference using bbregister (FreeSurfer) which implements boundary-based registration [97]. Co-registration was configured with twelve degrees of freedom to account for distortions remaining in the BOLD reference. Several confounding time-series were calculated based on the *preprocessed BOLD*: framewise displacement (FD), DVARS and three region-wise global signals. FD was computed using two formulations following Power (absolute sum of relative motions, [98]) and Jenkinson (relative root mean square displacement between affines, [96]). FD and DVARS are calculated for each functional run, both using their implementations in *Nipype* (following the definitions by [98]). The three global signals are extracted within the CSF, the WM, and the whole-brain masks. Additionally, a set of physiological regressors were extracted to allow for component-based noise correction (*CompCor*, [99]). Principal components are estimated after high-pass filtering the *preprocessed BOLD* time-series (using a discrete cosine filter with 128s cut-off) for the two *CompCor* variants: temporal (tCompCor) and anatomical (aCompCor). tCompCor components are then calculated from the top 2% variable voxels within the brain mask. For aCompCor, three probabilistic masks (CSF, WM and combined CSF+WM) are generated in anatomical space. The implementation differs from that of [99] in that instead of eroding the masks by 2 pixels on BOLD space, a mask of pixels that likely contain a volume fraction of GM is subtracted from the aCompCor masks. This mask is obtained by dilating a GM mask extracted from the FreeSurfer's aseg segmentation, and it ensures components are not extracted from voxels containing a minimal fraction of GM. Finally, these masks are resampled into BOLD space and binarized by thresholding at 0.99 (as in the original implementation). Components are also calculated separately within the WM and CSF masks. For each CompCor decomposition, the k components with the largest singular values are retained, such that the retained components' time series are sufficient to explain 50 percent of variance across the nuisance mask (CSF, WM, combined, or temporal). The remaining components are dropped from consideration. The head-motion estimates calculated in the correction step were also placed within the corresponding confounds file. The confound time series derived from head motion estimates and global signals were expanded with the inclusion of temporal

derivatives and quadratic terms for each [100]. Frames that exceeded a threshold of 0.5 mm FD or 1.5 standardized DVARS were annotated as motion outliers. Additional nuisance timeseries are calculated by means of principal components analysis of the signal found within a thin band (crown) of voxels around the edge of the brain, as proposed by [101]. The BOLD time-series were resampled onto the following surfaces (FreeSurfer reconstruction nomenclature): fsaverage, fsnative. The BOLD time-series were resampled onto the left/right-symmetric template “fsLR” using the Connectome Workbench [62]. Grayordinates files ([62]) containing 91k samples were also generated with surface data transformed directly to fsLR space and subcortical data transformed to 2 mm resolution MNI152NLin6Asym space. All resamplings can be performed with a single interpolation step by composing all the pertinent transformations (i.e. head-motion transform matrices, susceptibility distortion correction when available, and co-registrations to anatomical and output spaces). Gridded (volumetric) resamplings were performed using nitransforms, configured with cubic B-spline interpolation. Non-gridded (surface) resamplings were performed using mri_vol2surf (FreeSurfer).

Many internal operations of *fMRIPrep* use *Nilearn* 0.10.2 ([102], RRID:SCR_001362), mostly within the functional processing workflow. For more details of the pipeline, see the section corresponding to workflows in *fMRIPrep*’s documentation.

References

1. Chang, N. *et al.* BOLD5000, a public fMRI dataset while viewing 5000 visual images. *Sci Data* **6**, 49 (2019).
2. Hanke, M. *et al.* A studyforrest extension, simultaneous fMRI and eye gaze recordings during prolonged natural stimulation. *Sci Data* **3**, 160092 (2016).
3. Allen, E. J. *et al.* A massive 7T fMRI dataset to bridge cognitive neuroscience and artificial intelligence. *Nat Neurosci* **25**, 116–126 (2022).
4. Seeliger, K., Sommers, R. P., Güçlü, U., Bosch, S. E. & Gerven, M. A. J. van. A large single-participant fMRI dataset for probing brain responses to naturalistic stimuli in space and time. 687681 Preprint at https://doi.org/10.1101/687681 (2019).
5. Aliko, S., Huang, J., Gheorghiu, F., Meliss, S. & Skipper, J. I. A naturalistic neuroimaging database for understanding the brain using ecological stimuli. *Sci Data* **7**, 347 (2020).
6. Hebart, M. N. *et al.* THINGS-data, a multimodal collection of large-scale datasets for investigating object representations in human brain and behavior. *eLife* **12**, e82580 (2023).
7. Nishimoto, S. *et al.* Reconstructing Visual Experiences from Brain Activity Evoked by Natural Movies. *Current Biology* **21**, 1641–1646 (2011).
8. Wen, H. *et al.* Neural Encoding and Decoding with Deep Learning for Dynamic Natural Vision. *Cerebral Cortex* **28**, 4136–4160 (2018).
9. Zhou, M. *et al.* A large-scale fMRI dataset for human action recognition. *Sci Data* **10**, 415 (2023).
10. Boyle, J. A. *et al.* The Courtois project on neuronal modelling-first data release. in *26th annual meeting of the organization for human brain mapping* (2020).
11. Horikawa, T. & Kamitani, Y. Generic decoding of seen and imagined objects using hierarchical visual features. *Nat Commun* **8**, 15037 (2017).

12. Wang, J. *et al.* GIT: A Generative Image-to-text Transformer for Vision and Language. Preprint at https://doi.org/10.48550/arXiv.2205.14100 (2022).

13. Bartels, A. & Zeki, S. Functional brain mapping during free viewing of natural scenes. *Hum. Brain Mapp.* **21**, 75–85 (2004).

14. Konen, C. S. & Kastner, S. Representation of Eye Movements and Stimulus Motion in Topographically Organized Areas of Human Posterior Parietal Cortex. *Journal of Neuroscience* **28**, 8361–8375 (2008).

15. Press, W. A., Brewer, A. A., Dougherty, R. F., Wade, A. R. & Wandell, B. A. Visual areas and spatial summation in human visual cortex. *Vision Research* **41**, 1321–1332 (2001).

16. Schultz, J. & Pilz, K. S. Natural facial motion enhances cortical responses to faces. *Exp Brain Res* **194**, 465–475 (2009).

17. Yildirim, I., Wu, J., Kanwisher, N. & Tenenbaum, J. An integrative computational architecture for object-driven cortex. *Current Opinion in Neurobiology* **55**, 73–81 (2019).

18. Buccino, G. *et al.* Action Observation Activates Premotor and Parietal Areas in a Somatotopic Manner: An fMRI Study. in *Social Neuroscience* (Psychology Press, 2004).

19. Kret, M. E., Pichon, S., Grèzes, J. & de Gelder, B. Similarities and differences in perceiving threat from dynamic faces and bodies. An fMRI study. *NeuroImage* **54**, 1755–1762 (2011).

20. Lingnau, A. & Downing, P. E. The lateral occipitotemporal cortex in action. *Trends in Cognitive Sciences* **19**, 268–277 (2015).

21. Wurm, M. F. & Caramazza, A. Two ‘what’ pathways for action and object recognition. *Trends in Cognitive Sciences* **26**, 103–116 (2022).

22. Ahn, D., Kim, S., Hong, H. & Ko, B. C. STAR-Transformer: A Spatio-Temporal Cross Attention Transformer for Human Action Recognition. in 3330–3339 (2023).

23. Bertasius, G., Wang, H. & Torresani, L. Is Space-Time Attention All You Need for Video Understanding? in *Proceedings of the 38th International Conference on Machine Learning* 813–824 (PMLR, 2021).

24. Lin, J., Gan, C. & Han, S. TSM: Temporal Shift Module for Efficient Video Understanding. in 7083–7093 (2019).

25. Tong, Z., Song, Y., Wang, J. & Wang, L. VideoMAE: Masked Autoencoders are Data-Efficient Learners for Self-Supervised Video Pre-Training. *Advances in Neural Information Processing Systems* **35**, 10078–10093 (2022).

26. Wang, L. *et al.* Temporal Segment Networks: Towards Good Practices for Deep Action Recognition. in *Computer Vision – ECCV 2016* (eds. Leibe, B., Matas, J., Sebe, N. & Welling, M.) 20–36 (Springer International Publishing, Cham, 2016). doi:10.1007/978-3-319-46484-8_2.

27. Goyal, R. *et al.* The 'Something Something' Video Database for Learning and Evaluating Visual Common Sense. in 5842–5850 (2017).

28. Kay, W. *et al.* The Kinetics Human Action Video Dataset. Preprint at https://doi.org/10.48550/arXiv.1705.06950 (2017).

29. Miech, A. *et al.* HowTo100M: Learning a Text-Video Embedding by Watching Hundred Million Narrated Video Clips. in 2630–2640 (2019).

30. Monfort, M. *et al.* Moments in Time Dataset: One Million Videos for Event Understanding. *IEEE Trans. Pattern Anal. Mach. Intell.* **42**, 502–508 (2020).

31. Soomro, K., Zamir, A. R. & Shah, M. UCF101: A Dataset of 101 Human Actions Classes From Videos in The Wild. Preprint at https://doi.org/10.48550/arXiv.1212.0402 (2012).

32. Ho, J. *et al.* Imagen Video: High Definition Video Generation with Diffusion Models. Preprint at https://doi.org/10.48550/arXiv.2210.02303 (2022).

33. Singer, U. *et al.* Make-A-Video: Text-to-Video Generation without Text-Video Data. Preprint at https://doi.org/10.48550/arXiv.2209.14792 (2022).

34. Wu, J. Z. *et al.* Tune-A-Video: One-Shot Tuning of Image Diffusion Models for Text-to-Video Generation. in 7623–7633 (2023).

35. Ju, C., Han, T., Zheng, K., Zhang, Y. & Xie, W. Prompting Visual-Language Models for Efficient Video Understanding. in *Computer Vision – ECCV 2022* (eds. Avidan, S., Brostow, G., Cissé, M., Farinella, G. M. & Hassner, T.) 105–124 (Springer Nature Switzerland, Cham, 2022). doi:10.1007/978-3-031-19833-5_7.

36. Maaz, M., Rasheed, H., Khan, S. & Khan, F. S. Video-ChatGPT: Towards Detailed Video Understanding via Large Vision and Language Models. Preprint at https://doi.org/10.48550/arXiv.2306.05424 (2023).

37. Zhang, H., Li, X. & Bing, L. Video-LLaMA: An Instruction-tuned Audio-Visual Language Model for Video Understanding. Preprint at https://doi.org/10.48550/arXiv.2306.02858 (2023).

38. Liu, D. *et al.* Diffusion Action Segmentation. in 10139–10149 (2023).

39. Qing, Z. *et al.* MAR: Masked Autoencoders for Efficient Action Recognition. *IEEE Transactions on Multimedia* **26**, 218–233 (2024).

40. Zheng, C. *et al.* Deep Learning-based Human Pose Estimation: A Survey. *ACM Comput. Surv.* **56**, 11:1-11:37 (2023).

41. Misaki, M., Luh, W.-M. & Bandettini, P. A. Accurate decoding of sub-TR timing differences in stimulations of sub-voxel regions from multi-voxel response patterns. *NeuroImage* **66**, 623–633 (2013).

42. Prince, J. S. *et al.* Improving the accuracy of single-trial fMRI response estimates using GLMsingle. *eLife* **11**, e77599 (2022).

43. Wittkuhn, L. & Schuck, N. W. Dynamics of fMRI patterns reflect sub-second activation sequences and reveal replay in human visual cortex. *Nat Commun* **12**, 1795 (2021).

44. Fairhall, S. L., Albi, A. & Melcher, D. Temporal Integration Windows for Naturalistic Visual Sequences. *PLoS ONE* **9**, e102248 (2014).

45. Hasson, U., Yang, E., Vallines, I., Heeger, D. J. & Rubin, N. A Hierarchy of Temporal Receptive Windows in Human Cortex. *Journal of Neuroscience* **28**, 2539–2550 (2008).

46. Orlov, T. & Zohary, E. Object Representations in Human Visual Cortex Formed Through Temporal Integration of Dynamic Partial Shape Views. *J. Neurosci.* **38**, 659–678 (2018).

47. Doerig, A. *et al.* Semantic scene descriptions as an objective of human vision. (2022) doi:10.48550/ARXIV.2209.11737.

48. Kanwisher, N., Khosla, M. & Dobs, K. Using artificial neural networks to ask 'why' questions of minds and brains. *Trends in Neurosciences* **46**, 240–254 (2023).

49. Mineault, P., Bakhtiari, S., Richards, B. & Pack, C. Your head is there to move you around: Goal-driven models of the primate dorsal pathway. in *Advances in Neural Information Processing Systems* vol. 34 28757–28771 (Curran Associates, Inc., 2021).

50. Pitcher, D. & Ungerleider, L. G. Evidence for a Third Visual Pathway Specialized for Social Perception. *Trends in Cognitive Sciences* **25**, 100–110 (2021).

51. Chen, Z., Qing, J. & Zhou, J. H. Cinematic Mindscapes: High-quality Video Reconstruction from Brain Activity. Preprint at https://doi.org/10.48550/arXiv.2305.11675 (2023).

52. Cichy, R. M. *et al.* The Algonauts Project: A Platform for Communication between the Sciences of Biological and Artificial Intelligence. (2019) doi:10.48550/ARXIV.1905.05675.

53. Cichy, R. M. *et al.* The Algonauts Project 2021 Challenge: How the Human Brain Makes Sense of a World in Motion. (2021) doi:10.48550/ARXIV.2104.13714.

54. Esteban, O. *et al.* MRIQC: Advancing the automatic prediction of image quality in MRI from unseen sites. *PLoS ONE* **12**, e0184661 (2017).

55. Naselaris, T. *et al.* Cognitive Computational Neuroscience: A New Conference for an Emerging Discipline. *Trends in Cognitive Sciences* **22**, 365–367 (2018).

56. Yang, H., Zhang, S., Wu, Y., Li, Y. & Gu, S. Effective Ensemble of Deep Neural Networks Predicts Neural Responses to Naturalistic Videos. 2021.08.24.457581 Preprint at https://doi.org/10.1101/2021.08.24.457581 (2021).

57. Dumoulin, S. O. & Wandell, B. A. Population receptive field estimates in human visual cortex. *NeuroImage* **39**, 647–660 (2008).

58. Janik, R. A. & Olesik, M. A. bionn team solution preliminary write-up. (2021).

59. Nishimoto, S. Modeling movie-evoked human brain activity using motion-energy and space-time vision transformer features. 2021.08.22.457251 Preprint at https://doi.org/10.1101/2021.08.22.457251 (2021).

60. Watson, A. B. & Ahumada, A. J. Model of human visual-motion sensing. *J. Opt. Soc. Am. A, JOSAA* **2**, 322–342 (1985).

61. Esteban, O. *et al.* fMRIPrep: a robust preprocessing pipeline for functional MRI. *Nat Methods* **16**, 111–116 (2019).

62. Glasser, M. F. *et al.* The minimal preprocessing pipelines for the Human Connectome Project. *NeuroImage* **80**, 105–124 (2013).

63. Glasser, M. F. *et al.* A multi-modal parcellation of human cerebral cortex. *Nature* **536**, 171–178 (2016).

64. Robinson, E. C. *et al.* Multimodal surface matching with higher-order smoothness constraints. *NeuroImage* **167**, 453–465 (2018).

65. Gorgolewski, K. J. *et al.* The brain imaging data structure, a format for organizing and describing outputs of neuroimaging experiments. *Sci Data* **3**, 160044 (2016).

66. Gazzola, V. & Keysers, C. The Observation and Execution of Actions Share Motor and Somatosensory Voxels in all Tested Subjects: Single-Subject Analyses of Unsmoothed fMRI Data. *Cerebral Cortex* **19**, 1239–1255 (2009).

67. Le, A., Vesia, M., Yan, X., Crawford, J. D. & Niemeier, M. Parietal area BA7 integrates motor programs for reaching, grasping, and bimanual coordination. *Journal of Neurophysiology* **117**, 624–636 (2017).

68. Logothetis, N. K. & Sheinberg, D. L. Visual object recognition. *Annual Review of Neuroscience* **19**, 577–621 (1996).

69. Peeters, R. *et al.* The Representation of Tool Use in Humans and Monkeys: Common and Uniquely Human Features. *J. Neurosci.* **29**, 11523–11539 (2009).

70. Peeters, R. R., Rizzolatti, G. & Orban, G. A. Functional properties of the left parietal tool use region. *NeuroImage* **78**, 83–93 (2013).

71. Rizzolatti, G. & Sinigaglia, C. The functional role of the parieto-frontal mirror circuit: interpretations and misinterpretations. *Nat Rev Neurosci* **11**, 264–274 (2010).

72. Silver, M. A. & Kastner, S. Topographic maps in human frontal and parietal cortex. *Trends in Cognitive Sciences* **13**, 488–495 (2009).

73. VanRullen, R. & Thorpe, S. J. The Time Course of Visual Processing: From Early Perception to Decision-Making. *Journal of Cognitive Neuroscience* **13**, 454–461 (2001).

74. Julian, J. B., Fedorenko, E., Webster, J. & Kanwisher, N. An algorithmic method for functionally defining regions of interest in the ventral visual pathway. *NeuroImage* **60**, 2357–2364 (2012).

75. Wang, L., Mruczek, R. E. B., Arcaro, M. J. & Kastner, S. Probabilistic Maps of Visual Topography in Human Cortex. *Cerebral Cortex* **25**, 3911–3931 (2015).

76. Georgieva, S., Peeters, R., Kolster, H., Todd, J. T. & Orban, G. A. The Processing of Three-Dimensional Shape from Disparity in the Human Brain. *Journal of Neuroscience* **29**, 727–742 (2009).

77. Smith, A. T., Greenlee, M. W., Singh, K. D., Kraemer, F. M. & Hennig, J. The Processing of First- and Second-Order Motion in Human Visual Cortex Assessed by Functional Magnetic Resonance Imaging (fMRI). *J. Neurosci.* **18**, 3816–3830 (1998).

78. Mineroff, Z., Blank, I. A., Mahowald, K. & Fedorenko, E. A robust dissociation among the language, multiple demand, and default mode networks: Evidence from inter-region correlations in effect size. *Neuropsychologia* **119**, 501–511 (2018).

79. Adelson, E. H. & Bergen, J. R. Spatiotemporal energy models for the perception of motion. *J. Opt. Soc. Am. A, JOSAA* **2**, 284–299 (1985).

80. Born, R. T. & Bradley, D. C. Structure and Function of Visual Area Mt. *Annual Review of Neuroscience* **28**, 157–189 (2005).

81. Nishimoto, S. & Gallant, J. L. A Three-Dimensional Spatiotemporal Receptive Field Model Explains Responses of Area MT Neurons to Naturalistic Movies. *J. Neurosci.* **31**, 14551–14564 (2011).

82. Kamitani, Y. & Tong, F. Decoding seen and attended motion directions from activity in the human visual cortex. *Curr Biol* **16**, 1096–1102 (2006).

83. Roe, A. W. *et al.* Toward a Unified Theory of Visual Area V4. *Neuron* **74**, 12–29 (2012).

84. Naselaris, T., Kay, K. N., Nishimoto, S. & Gallant, J. L. Encoding and decoding in fMRI. *NeuroImage* **56**, 400–410 (2011).

85. Esteban, O. *et al.* fMRIPrep: a robust preprocessing pipeline for functional MRI. Zenodo https://doi.org/10.5281/zenodo.7430291 (2022).

86. Esteban, Oscar *et al.* nipy/nipype: 1.8.3. Zenodo https://doi.org/10.5281/ZENODO.596855 (2022).

87. Gorgolewski, K. *et al.* Nipype: A Flexible, Lightweight and Extensible Neuroimaging Data Processing Framework in Python. *Front. Neuroinform.* **5**, (2011).

88. Tustison, N. J. *et al.* N4ITK: Improved N3 Bias Correction. *IEEE Transactions on Medical Imaging* **29**, 1310–1320 (2010).

89. Avants, B. B., Epstein, C. L., Grossman, M. & Gee, J. C. Symmetric diffeomorphic image registration with cross-correlation: Evaluating automated labeling of elderly and neurodegenerative brain. *Medical Image Analysis* **12**, 26–41 (2008).

90. Zhang, Y., Brady, M. & Smith, S. Segmentation of brain MR images through a hidden Markov random field model and the expectation-maximization algorithm. *IEEE Transactions on Medical Imaging* **20**, 45–57 (2001).

91. Dale, A. M., Fischl, B. & Sereno, M. I. Cortical Surface-Based Analysis. *NeuroImage* **9**, 179–194 (1999).

92. Klein, A. *et al.* Mindboggling morphometry of human brains. *PLoS Comput Biol* **13**, e1005350 (2017).

93. Ciric, R. *et al.* TemplateFlow: FAIR-sharing of multi-scale, multi-species brain models. *Nat Methods* **19**, 1568–1571 (2022).

94. Fonov, V., Evans, A., McKinstry, R., Almli, C. & Collins, D. Unbiased nonlinear average age-appropriate brain templates from birth to adulthood. *NeuroImage* **47**, S102 (2009).

95. Evans, A. C., Janke, A. L., Collins, D. L. & Baillet, S. Brain templates and atlases. *NeuroImage* **62**, 911–922 (2012).

96. Jenkinson, M., Bannister, P., Brady, M. & Smith, S. Improved Optimization for the Robust and Accurate Linear Registration and Motion Correction of Brain Images. *NeuroImage* **17**, 825–841 (2002).

97. Greve, D. N. & Fischl, B. Accurate and robust brain image alignment using boundary-based registration. *NeuroImage* **48**, 63–72 (2009).

98. Power, J. D. *et al.* Methods to detect, characterize, and remove motion artifact in resting state fMRI. *NeuroImage* **84**, 320–341 (2014).

99. Behzadi, Y., Restom, K., Liau, J. & Liu, T. T. A component based noise correction method (CompCor) for BOLD and perfusion based fMRI. *NeuroImage* **37**, 90–101 (2007).

100. Satterthwaite, T. D. *et al.* An improved framework for confound regression and filtering for control of motion artifact in the preprocessing of resting-state functional connectivity data. *NeuroImage* **64**, 240–256 (2013).

101. Patriat, R., Reynolds, R. C. & Birn, R. M. An improved model of motion-related signal changes in fMRI. *NeuroImage* **144**, 74–82 (2017).

102. Abraham, A. *et al.* Machine learning for neuroimaging with scikit-learn. *Frontiers in Neuroinformatics* **8**, (2014).
