# Supplementary material for: Modeling short visual events through the BOLD moments video fMRI dataset and metadata
Source: Nat Commun. 2024 Jul 24;15:6241. doi: 10.1038/s41467-024-50310-3 (PMC11269733; doi:10.1038/s41467-024-50310-3)
Supplement: Supplementary file 3 — Reporting Summary [file 41467_2024_50310_MOESM3_ESM.pdf]

nature portfolio

Corresponding author(s): Benjamin Lahner

Last updated by author(s): May 18, 2024

Reporting Summary

Nature Portfolio wishes to improve the reproducibility of the work that we publish. This form provides structure for consistency and transparency in reporting. For further information on Nature Portfolio policies, see our Editorial Policies and the Editorial Policy Checklist.

Statistics

For all statistical analyses, confirm that the following items are present in the figure legend, table legend, main text, or Methods section.

n/a	Confirmed	
☐	☒	The exact sample size (*n*) for each experimental group/condition, given as a discrete number and unit of measurement
☐	☒	A statement on whether measurements were taken from distinct samples or whether the same sample was measured repeatedly
☐	☒	The statistical test(s) used AND whether they are one- or two-sided<br>*Only common tests should be described solely by name; describe more complex techniques in the Methods section.*
☐	☒	A description of all covariates tested
☐	☒	A description of any assumptions or corrections, such as tests of normality and adjustment for multiple comparisons
☐	☒	A full description of the statistical parameters including central tendency (e.g. means) or other basic estimates (e.g. regression coefficient) AND variation (e.g. standard deviation) or associated estimates of uncertainty (e.g. confidence intervals)
☐	☒	For null hypothesis testing, the test statistic (e.g. *F*, *t*, *r*) with confidence intervals, effect sizes, degrees of freedom and *P* value noted<br>*Give P values as exact values whenever suitable.*
☒	☐	For Bayesian analysis, information on the choice of priors and Markov chain Monte Carlo settings
☒	☐	For hierarchical and complex designs, identification of the appropriate level for tests and full reporting of outcomes
☐	☒	Estimates of effect sizes (e.g. Cohen's *d*, Pearson's *r*), indicating how they were calculated

Our web collection on statistics for biologists contains articles on many of the points above.

Software and code

Policy information about availability of computer code

Data collection	For data collection, we use MATLAB version 2017 with Psychtoolbox-3 (http://psychtoolbox.org/) to present stimuli to the subjects in the MRI. scanner.
Data analysis	We preprocess the (f)MRI data using the open source fMRIPrep 20. 2.1 software. We additionally use MATLAB (version 2017), SPM1 2, and Python 3 for additional data preparation and analyses. Our Code Availability section is the following:<br>Code used in this manuscript have been provided alongside the data in the OpenNeuro database under accession code ds005165 [https://openneuro.org/datasets/ds005165]. The TSM ResNet50 model training code is available here [https://github.com/pbw-Berwin/M4-pretrained]. Starter code demonstrating basic usage of the dataset is available here [https://github.com/blahner/BOLDMomentsDataset].

For manuscripts utilizing custom algorithms or software that are central to the research but not yet described in published literature, software must be made available to editors and reviewers. We strongly encourage code deposition in a community repository (e.g. GitHub). See the Nature Portfolio guidelines for submitting code & software for further information.

Data

Policy information about availability of data

All manuscripts must include a data availability statement. This statement should provide the following information, where applicable:

- Accession codes, unique identifiers, or web links for publicly available datasets
- A description of any restrictions on data availability
- For clinical datasets or third party data, please ensure that the statement adheres to our policy

Our Data Availability statement is the following:
The (f)MRI data, stimulus metadata, and TSM ResNet50 model weights generated in this study have been deposited in the OpenNeuro database under accession code ds005165 [https://openneuro.org/datasets/ds005165]. The original video stimuli can be accessed from the Moments in Time, Multi-Moments in Time, and Memento10k datasets, available at the following links [http://moments.csail.mit.edu/] and [http://memento.csail.mit.edu/]. Source data are provided with this paper.

Research involving human participants, their data, or biological material

Policy information about studies with human participants or human data. See also policy information about sex, gender (identity/presentation), and sexual orientation and race, ethnicity and racism.

Reporting on sex and gender	While we record sex (6 self-reported female), we do not use this information in our analyses. We are studying the visual perception of short videos and have no reason to believe sex or gender plays a substantial role.
Reporting on race, ethnicity, or other socially relevant groupings	We did not perform any groupings of subjects, using race, ethnicity, socially relevant groupings, or otherwise.
Population characteristics	Participants self-reported their age (27.01 +/- 3.96 years mean+/- STD) and confirmed normal or corrected-to-normal vision.
Recruitment	Participants were recruited locally around the university through email messaging. This recruitment may bias participants towards a certain age group and social status. However, such biases are not thought to have any significant impact on visual processing.
Ethics oversight	The Institutional Review Board of Massachusetts Institute of Technology approved this study (approval code: 1510287948).

Note that full information on the approval of the study protocol must also be provided in the manuscript.

Field-specific reporting

Please select the one below that is the best fit for your research. If you are not sure, read the appropriate sections before making your selection.

☒ Life sciences ☐ Behavioural & social sciences ☐ Ecological, evolutionary & environmental sciences

For a reference copy of the document with all sections, see nature.com/documents/nr-reporting-summary-flat.pdf

Life sciences study design

All studies must disclose on these points even when the disclosure is negative.

Sample size	We use a sample size of n=l0 human participants. No prior sample size calculation was performed. We chose this sample size to achieve a large amount of within-subject and between-subject brain measurements with multiple stimulus repetitions for increased signal. The large amount of within-subject measurements allows in-depth subject-specific analyses. The repeated experiment across multiple subjects allow for replication of findings across subjects and power for group-level analyses. Our sample size is comparable to other large-scale fMRI datasets.
Data exclusions	We did not exclude any data from analysis.
Replication	We provide the original data, its derivatives, and code to reproduce our preprocessing and analysis results, including plots and statistics. We have internally run the code multiple times to verify the same results.
Randomization	Experimental groups were not relevant in this study. Our study aims did not include differences between group.
Blinding	Blinding was not relevant to this study as this study did not incorporate multiple experimental groups.

Reporting for specific materials, systems and methods

We require information from authors about some types of materials, experimental systems and methods used in many studies. Here, indicate whether each material, system or method listed is relevant to your study. If you are not sure if a list item applies to your research, read the appropriate section before selecting a response.

Materials & experimental systems

n/a	Involved in the study
x	Antibodies
x	Eukaryotic cell lines
x	Palaeontology and archaeology
	x Animals and other organisms
x	Clinical data
x	Dual use research of concern
x	Plants

Methods

n/a	Involved in the study
x	ChIP-seq
x	Flow cytometry
	x MRI-based neuroimaging

Animals and other research organisms

Policy information about studies involving animals; ARRIVE guidelines recommended for reporting animal research, and Sex and Gender in Research

Laboratory animals	This study does not involve laboratory animals.
Wild animals	This study does not involve wild animals.
Reporting on sex	Of our ten participants, six identified as female. Results apply to both sexes, and sex was not considered in study design. We do not perform any sex-based analyses. Our study examines visual perception of short videos in humans, which is not believed to be influenced by sex.
Field-collected samples	This study does not involve samples collected from the field.
Ethics oversight	The experiment was conducted in accordance with the Declaration of Helsinki and approved by the local ethics committee (Institutional Review Board of Massachusetts Institute of Technology, approval code: 1510287948).

Note that full information on the approval of the study protocol must also be provided in the manuscript.

Plants

Seed stocks	*Report on the source of all seed stocks or other plant material used. If applicable, state the seed stock centre and catalogue number. If plant specimens were collected from the field, describe the collection location, date and sampling procedures.*
Novel plant genotypes	*Describe the methods by which all novel plant genotypes were produced. This includes those generated by transgenic approaches, gene editing, chemical/radiation-based mutagenesis and hybridization. For transgenic lines, describe the transformation method, the number of independent lines analyzed and the generation upon which experiments were performed. For gene-edited lines, describe the editor used, the endogenous sequence targeted for editing, the targeting guide RNA sequence (if applicable) and how the editor was applied.*
Authentication	*Describe any authentication procedures for each seed stock used or novel genotype generated. Describe any experiments used to assess the effect of a mutation and, where applicable, how potential secondary effects (e.g. second site T-DNA insertions, mosiacism, off-target gene editing) were examined.*

Magnetic resonance imaging

Experimental design

Design type	We performed three independent experiments, (1) resting state, (2) task-based block design localizer, and (3) task-based event-related design.
Design specifications	(1) 5 runs of resting state scans were collected, with each run lasting 6 minutes and 18 seconds. (2) 5 runs of the task-based block design localizer experiment were collected, with each run lasting 7 minutes and 30 seconds. Each run consisted of 5 null (gray screen) blocks and 20 stimulus presentation blocks, with each block lasting 18 seconds. (3) The task-based event-related experiment consisted of 52 runs spread out over 4 sessions (13 runs per session). Within each session, 10 runs presented a "training" subset of stimuli ("training" runs) and 3 sessions presented a "testing" subset of stimuli ("testing" runs). The "training" runs consisted of 100 trials and "testing" runs consisted of 113 trials. Besides the stimulus subset presented and number of trials, the "training" and "testing" runs were identical. Each run consisted of 75%/25% stimulus/null trials where each trial was 4 seconds in length. With the 4 second trial, the stimulus was 3 seconds in duration, resulting in an interval of 1 second between stimulus presentations.
Behavioral performance measures	The block design localizer experiment used a one-back vigilance task. Subject accuracy was 0.941 +/- 0.011 (mean± SD). The event-related main experiment incorporated a vigilance task to press a button on null trials, where no stimulus was presented and the fixation cross dimmed to a darker color. Subject accuracy was 0.964 +/- 0.014 (mean± SD).

Acquisition

Imaging type(s)

functional, structural

Field strength

3T

Sequence & imaging parameters

The MRI data were acquired with a 3T Trio Siemens scanner using a 32-channel head coil. During the experimental runs, T2*-weighted gradient-echo echo-planar images (EPI) were collected (TR= 1750 ms, TE= 30 ms, flip angle= 71°, FOV read= 190 mm, FOV phase= 100%, bandwidth = 2268 Hz/Px, resolution= 2.5 x 2.5 x 2.5 mm, slice gap= 10%, slices= 54, multi-band acceleration factor= 2, ascending interleaved acquisition). Additionally, a Tl-weighted image (TR= 1900 ms, TE= 2.52 ms, flip angle= 9°, FOV read= 256mm, FOV phase= 100%, bandwidth= 170 Hz/px, resolution= 1.0 x 1.0 x 1.0 mm, slices= 176 sagittal slices, multi-slice mode= single shot, ascending) and T2-weighted image (TR= 7970ms, TE= 120 ms, flip angle= 90°, FOV read= 256 mm, FOV phase= 100%, bandwidth= 362 Hz/Px, resolution= 1.0 x 1.0 x 1.1 mm, slice gap= 10%, slices= 128, multi-slice mode= interleaved, ascending) were obtained as high-resolution anatomical references. We acquired resting state and functional localizer data using acquisition parameters identical to the main experimental runs.

Area of acquisition

Whole Brain

Diffusion MRI ☐ Used ☒ Not used

Preprocessing

Preprocessing software

We preprocess the functional data using fMRIPrep 20.2.1. We perform slice-time correction, co-registration, and normalization. The experimental runs for the functional localizer were smoothed with a 9mm kernel. The experimental runs for the main experiment were not smoothed. Concerning brain extraction and segmentation, we reproduce relevant text from fMRIPrep output here, "The Tl-weighted (Tlw) image was corrected for intensity non-uniformity (INU) with N4BiasFieldCorrection (Tustison et al., 2010), distributed with ANTs 2.3.3 (Avants et al., 2008, RRID:SCR_004757), and used as Tlw-reference throughout the workflow. The Tlw-reference was then skull-stripped with a Nipype implementation of the antsBrainExtraction.sh workflow (from ANTs), using OASIS30ANTs as target template. Brain tissue segmentation of cerebrospinal fluid (CSF), white-matter (WM) and gray-matter (GM) was performed on the brain-extracted Tlw using fast (FSL 5.0.9, RRID:SCR_002823, Zhang et al., 2001). Brain surfaces were reconstructed using recon-all (FreeSurfer 6.0.1, RRID:SCR_001847, Dale et al., 1999), and the brain mask estimated previously was refined with a custom variation of the method to reconcile ANTs-derived and FreeSurfer-derived segmentations of the cortical gray-matter of Mindboggle (RRID:SCR_002438, Klein et al., 2017)."

Normalization

We performed a non-linear volume-based spatial normalization to the standard MNl152NLin2009cAsym space. We used antsRegistration (ANTs 2.3.3) to perform this procedure.

Normalization template

ICBM 152 Nonlinear Asymmetrical template version 2009c

Noise and artifact removal

Please read the following relevant text from fMRIPrep output: "Head-motion parameters with respect to the BOLD reference (transformation matrices, and six corresponding rotation and translation parameters) are estimated before any spatiotemporal filtering using mcflirt (FSL 5.0.9, Jenkinson et al., 2002). [...] Several confounding time-series were calculated based on the preprocessed BOLD: framewise displacement (FD), DVARS and three region-wise global signals. [...] Additionally, a set of physiological regressors were extracted to allow for component-based noise correction (CompCor, Behzadi et al., 2007). [...] For each CompCor decomposition, the k components with the largest singular values are retained, such that the retained components' time series are sufficient to explain 50 percent of variance across the nuisance mask (CSF, WM, combined, or temporal). The remaining components are dropped from consideration. The head-motion estimates calculated in the correction step were also placed within the corresponding confounds file. The confound time series derived from head motion estimates and global signals were expanded with the inclusion of temporal derivatives and quadratic terms for each (Satterthwaite et al., 2013). Frames that exceeded a threshold of 0.5 mm FD or 1.5 standardised DVARS were annotated as motion outliers." We use a general linear model to estimate the betas. For the functional localizer experiment, we include motion and run regressors as regressors of no interest. We convolve all regressors with a canonical HRF to estimate the betas, and we account for unmodeled neuronal activity and aliased biorhythms with an autoregressive AR(l) model. For the main experiment, a weighted combination of simple Finite Impulse Response (FIR) basis functions. We modeled the BOLD response with respect to each video onset from 1 to 9 seconds in 1 second steps (corresponding to the resolution of the resampled time series). Within this time interval the voxel-wise time course of activation was high-pass filtered (removing signal with f<l/128 Hz) and serial correlations due to aliased biorhythms or unmodelled neuronal activity were accounted for using an autoregressive AR(l) model.

Volume censoring

We did not censor any volumes.

Statistical modeling & inference

Model type and settings

We employ a variety of analyses in this manuscript. A mass univariate analysis correlates (Spearman's R) a vector of stimulus memorability scores with stimulus-evoked beta estimates at each voxel. Multivariate RSA analyses use searchlight (sphere of 4 voxel radius) to extract vectors of beta estimates and compute pairwise distances (1-Pearson R) to create a representational dissimilarity matrix (ROM) at each voxel. These RDMs were subsequently correlated with RDMs representing stimuli semantic meta data (features extracted from language models). Predictive analyses employed neural networks to extract stimulus features at different layers, representing hypotheses of visual processing, and trained a linear model to predict brain activity (encoding model procedure).

Effect(s) tested | ANOVA and factorial designs were not used.

Specify type of analysis: ☐ Whole brain ☐ ROI-based ☒ Both

Anatomical location(s) | We functionally defined categorically-selective ROIs for each subject using an independent functional localizer experiment. We additionally used ROI definitions provided by Wang and colleagues (2015) and Anatomical location(s) Glasser and colleagues (2016). For these ROIs, we resliced the provided ROI volume in MNI152 space into the appropriate volume dimensions in our experiment. The resulting ROI mask was used to extract brain activity within the ROI.

Statistic type for inference | voxel-wise

(See Eklund et al. 2016)

Correction | For whole brain analyses, we used FDR correction (assuming positive correlation) to adjust the p-values. For ROI analyses, we used bonferroni correction (n=22).

Models & analysis

n/a	Involved in the study
☒	☐ Functional and/or effective connectivity
☒	☐ Graph analysis
☐	☒ Multivariate modeling or predictive analysis

Multivariate modeling and predictive analysis | We trained a Deep Neural Network (TSM model with ResNet50 backbone) on an action recognition task using the Multi-Moments in Time dataset. We ensured that there was no overlap between the training data and BOLD Moments stimuli. We then performed inference on the network, extracted features at each block, and used PCA to reduce dimensionality. We trained a linear model to fit the extracted features to the beta estimates and subsequently predict beta estimates of the test data. Similar procedures were followed in our other encoding model analyses. We additionally used a searchlight method (spherical, radius of 4 voxels) to compute representational dissimilarity matrices (RDMs) at each voxel. RDMs were computed using 1-Pearson R correlation distance between pairs of vectors of beta estimates for voxels within the searchlight from each experimental condition.
